# Residues of plant protection products in grey partridge eggs in French cereal ecosystems

**DOI:** 10.1007/s11356-016-6093-7

**Published:** 2016-02-03

**Authors:** Elisabeth Bro, James Devillers, Florian Millot, Anouk Decors

**Affiliations:** National Game and Wildlife Institute (ONCFS), Research Department, Saint Benoist, 78610 Auffargis, France; Centre de Traitement de l’Information Scientifique, 3 chemin de la Gravière, 69140 Rillieux La Pape, France

**Keywords:** Bird, Egg, Exposure route, Farmland, Residue analysis, Pesticide

## Abstract

The contamination of the eggs of farmland birds by currently used plant protection products (PPPs) is poorly documented despite a potential to adversely impact their breeding performance. In this context, 139 eggs of 52 grey partridge *Perdix perdix* clutches, collected on 12 intensively cultivated farmlands in France in 2010–2011, were analysed. Given the great diversity of PPPs applied on agricultural fields, we used exploratory GC/MS-MS and LC/MS-MS screenings measuring ca. 500 compounds. The limit of quantification was 0.01 mg/kg, a statutory reference. A total of 15 different compounds were detected in 24 clutches. Nine of them have been used by farmers to protect crops against fungi (difenoconazole, tebuconazole, cyproconazole, fenpropidin and prochloraz), insects (lambda-cyhalothrin and thiamethoxam/clothianidin) and weeds (bromoxynil and diflufenican). Some old PPPs were also detected (fipronil(+sulfone), HCH(α,β,δ isomers), diphenylamine, heptachlor(+epoxyde), DDT(Σisomers)), as well as PCBs(153, 180). Concentrations ranged between <0.01 and 0.05 mg/kg but reached 0.067 (thiamethoxam/clothianidin), 0.11 (heptachlor + epoxyde) and 0.34 (fenpropidin) mg/kg in some cases. These results testify an actual exposure of females and/or their eggs to PPPs in operational conditions, as well as to organochlorine pollutants or their residues, banned in France since several years if not several decades, that persistently contaminate the environment.

Routes of exposure, probability to detect a contamination in the eggs, and effects on egg/embryo characteristics are discussed with regard to the scientific literature.

**Electronic supplementary material:**

The online version of this article (doi:10.1007/s11356-016-6093-7) contains supplementary material, which is available to authorized users.

## Introduction

The most recent European Red List reviewing the conservation status of all European species reports that only 48 % of bird species associated with agricultural ecosystems are classified within the status “*least concern*”—to be compared to 80 % for all bird species (BirdLife International 2015a). The rate of biodiversity loss in farmlands is therefore still worrying, despite target 3 of the European Union strategic plan for biodiversity aims to “increase the contribution of agriculture and forestry to maintaining and enhancing biodiversity” by 2020 (European Commission 2011).

Farmland bird species have suffered from severe historical declines (European Bird Census Council 2015). It is well established that intensification of farming practices and associated changes in habitat were the main drivers of this decline, through direct and indirect effects (e.g., Benton et al. 2002; Chamberlain et al. 2000; Donald et al. 2001, 2006; Evans 2004; Robinson and Sutherland 2002; and references therein). Farmland birds continue to decline today (Comolet-Tirman et al. 2015; European Bird Census Council 2015), as a result of various threats including illegal hunting, climate change and severe weather, interaction with invasive species, changes in land-use practices (intensification of agriculture and land abandonment), pollution, etc. (BirdLife International 2015a). Farming practices and habitat changes are, however, still major drivers of bird decline (BirdLife 2015a; Eglington and Pearce-Higgins 2012; Stoate et al. 2009). Recent works suggest that the agricultural use of actual plant protection products (PPPs) still contributes to the ongoing decline of avian populations (Bro et al. 2010; Geiger et al. 2010; Hallmann et al. 2014; Mineau and Whiteside 2013; van der Sluijs et al. 2015; van Lexmond et al. 2015). Indeed, although bird ecotoxicology has been poorly documented compared to other taxa (Köhler and Triebskorn 2013), it is admitted from field surveys, correlative data analysis and laboratory experiments that some active substances (ASs) can affect the survival of birds and their breeding success through different mechanisms.

Adverse effects may occur indirectly through a reduction in food abundance (weeds, seeds and invertebrates) resulting in a poor breeding success (e.g., Boatman et al. 2004; Hart et al. 2006; Rands 1985). They may also occur directly by oral, air or contact contamination. Intoxication can then lead to death or to sublethal behavioural or immune disorders that can favour the death (cf. Garg et al. 2004; Lopez-Antia et al. 2013, 2015; Millot et al. 2015; and ref therein), as well as reproductive disorders such as anomalies in courtship behaviour (Fernie et al. 2003; Ottinger et al. 2008); anatomical anomalies of gonads (Bauer 1985); decrease in chick production rate through reduced clutch size, clutch abandonment, reduced fertility, teratogenicity or other effects such as eggshell thinning (Fernie et al. 2003; Kamata et al. 2010; Kitulagodage et al. 2011; Lopez-Antia et al. 2015; Maci and Arias 1987; Mendenhall et al. 1983; Mineau 2005); reduced chick survival and poor chick condition (Bhaskar et al. 2012; Kitulagodage et al. 2011; Lopez-Antia et al. 2015; Mineau 2005; Nitu et al. 2012; Uggini et al. 2012). Intergenerational effects are also observed (Bauer 1985; Fernie et al. 2003).

However, such effects are documented for only a small number of ASs that are currently used. In addition, most of the above very interesting cited works are lab studies. They provide useful information but no firm conclusion that such effects actually occur in the “real world”. Additional field works are therefore needed to consolidate the results. The first requirement is the proof of a real contamination of the birds and their eggs in operational conditions. There is a large body of scientific literature on this subject, but few studies deal with current pesticide ASs and with herbivorous-granivorous-insectivorous farmland birds. In this context, this study contributes to fill this gap of knowledge by providing field data for a typical farmland bird, the grey partridge (*Perdix perdix*). The ecology of this ground nesting bird makes it a suitable focal species: (i) it mainly lays its clutches in crops, with a preference for winter cereals (Bro et al. 2013) and (ii) it feeds on a variety of items including sprouts, grains and seeds of both cultivated and weed plants, and invertebrates. In addition, the species is of conservation concern in several European states (UK, Sotherton et al. 2014; France—“Centre” area, CSRPN 2013; Switzerland, Keller et al. 2010; etc.) due to its ongoing population decline and/or range contraction (BirdLife International 2015b). The management of this game species benefitted from a large amount of research throughout the world (e.g., review of Sotherton et al. 2014 for UK; e.g., Bro et al. 2000a, b, 2001, 2004, 2013 for France), but relatively little attention was paid to field ecotoxicology so far.

## Methods

### Egg collection and storage

We collected failed eggs from hatched, destroyed and deserted clutches of radiotagged grey partridge females (Bro et al. 2015). They were put in quail egg boxes and stored in the dark at −20 °C.

### Examination of egg and embryo status

Intact failed eggs were opened in the lab to examine their content. They were classified as “infertile” (no germinal disc observed, which includes embryos at development stage ≤1–2 days; Bro et al. 2013; McCabe and Hawkins 1946), “dead embryo” and “undetermined” (when we were not certain of the status of the egg, such as in the case of rotten eggs). We looked by eye for macroscopical deformities of embryos ≥15 days old (bill, skull, eye or leg defects; Ludwig et al. 1996). Eggshell thickness was measured to the nearest 0.001 mm by the same investigator using a digital micrometer (IP65 0–25 mm, Mitutoyo, Japan). Measures were done at the equator region after a careful separation of the inner membranes.

### Residue analysis

#### Quality assurance

Residue analyses were performed by Phytocontrol (Nîmes, France). The laboratory is accredited by the French Committee of Accreditation (COFRAC) for the research and quantification of pesticides in foodstuffs (no. 1–1904, COFRAC 2010). It works in compliance with the international standard ISO/IEC 17025 and according to the LAB GTA 26 99-2 program. In addition, the laboratory is certified ISO 14001 by the French Agency of Normalisation (AFNOR).

The methods were validated using several criteria: recovery rates, repeatability, reproducibility, specificity and linearity (SANCO/12571/2013, European Commission / Health & Consumer Protection Directorate 2013).

#### Multi-residue analyses

##### Sample preparation

Samples were prepared using a QuEChERS protocol which couples an extraction method of pesticides using a solvent and a clean-up method using purification salts adapted to the matrix and to the substances. We used QuEChERS no. NF EN 15662.

##### Extraction

Ten grams of the whole egg was mixed with 10 mL of pure water and 10 mL of acetonitrile. Acetonitrile was used as the extraction solvent because of its effectiveness to remove polar components such as sugars, lipids, organic acids, sterols, proteins, pigments and excess water. Salts (4 g MgSO_4_, 1 g NaCl, citrate buffer at pH = 5–5.5) were added, and the mixture was vigorously shaken for 10 min and then centrifuged at 15 °C for 5 min at 3000*×g* to separate the solid and liquid phases.

##### Purification

An aliquot of the supernatant (5 mL) was purified using a dispersive solid phase extraction (d-SPE) involving salts containing 900 mg of anhydrous MgSO_4_, 300 mg of PSA and 300 mg of C18. After a vigorous shaking for 10 min, mixtures were centrifuged at 15 °C for 5 min at 3000*×g*.

The supernatant (8 mL) was split into two fractions of 4 mL and evaporated under a nitrogen stream. One fraction was acidified with 5 % formic acid solution and redissolved in 0.2 mL of acetonitrile for GC/MS-MS analysis. The second fraction was redissolved in 0.2 mL of mobile phase (0.1 % acetic acid in water/acetonitrile, 50/50 *v*/*v*) for LC/MS-MS analysis.

##### Identification and quantification

Five microliters and 2 μL were injected in LC and GC, respectively. Compounds were identified and quantified with a triple quadrupole tandem (QqQ) mass spectrometry (electrospray source, pos and neg). The use of QqQ analyzers improves the sensitivity and the selectivity of the analysis. Each compound was characterised by its retention time, a quantitation transition, a confirming transition and the ratio between the signals of these transitions. For linearity, *R* values ranged between 70 and 120 %.

Residues of ca. 500 compounds were measured using both LC/MS-MS and GC/MS-MS screenings (Online Resource 1). Compounds were ASs and/or their isomers and/or their metabolites. The recovery yields of all compounds varied between 70 and 120 %, with a coefficient of variation of 20 %.

Of the ASs we listed as used by the farmers in our 12 study sites in spring and summer 2010–2011, 85.5 % were measured (Online Resource 2, Bro et al. 2015). The remaining ASs were not measured either because they were not proposed in routine by the laboratory or because they needed costly specific analyses. Their inclusion would have severely exceeded our budget for pesticide residue analysis.

##### Intrumentation

For GC/MS-MS, analyses were carried out using GC/MS-MS Scion (Bruker). Quantification was performed with a workstation from Bruker. For LC/MS-MS, analyses were carried out using a Shimadzu 8040. Quantification was performed with Labsolution from Shimadzu.

##### Quality control

In each batch of samples, two controls were included: a reagent blank consisting of a vial containing only solvent extract and an internal laboratory quality control (QC, concentration 100 ppb) consisting of a spiked matrix with a mix of pesticides. The batch analyses were considered valid when the values of the analytes in the QC were within a range of 70–120 % of the theoretical value.

##### Analytical performance

The analytical methods used allowed to reach a limit of quantification (LoQ) of 0.01 mg/kg for almost all compounds—LoQ was lower for fipronil(+sulfone): 0.005 mg/kg, and higher for flonicamide(+TNFA + TNFG) and TNFG: 0.05 mg/kg (Online Resource 1). The limit of detection (LoD) was approximatively half of LoQ. The value of 0.01 mg/kg is the default value for maximum residue levels (MRLs)^1^ when no specific MRL is set out for a given product (Regulation (EC) No. 396/2005, article 18 1.(b)).

### Selection of clutches and eggs for analyses

We analysed intact failed eggs. This is a common practice because this sampling is non-invasive. The drawback is the bias in the sample, which limits the cause to effect and other quantitative interpretation of the results (see Discussion), but it does not weaken an exploratory analysis of egg contamination. Another common practice is to analyse the first or the second egg laid (e.g., Eng et al. 2014) to limit the variability in the results. However, in our case, this was neither possible because we did not know the laying sequence (the location of a nest is only known once the clutch is completed and incubation is initiated), nor desirable. Indeed, as much as 18 eggs—sometimes more—can be laid within ca. 20–30 days. Thus, all of them are not likely to be exposed to an AS following its application (see Bro et al. 2015). As a consequence, a non-positive result obtained on one egg cannot be extrapolated to the whole clutch. The analysis of several eggs is then required to maximise the probability to detect a contamination. In this context, we sometimes pooled a few eggs in a same sample, trying to find the best compromise between the risk of a potential dilution effect on the one hand and the funds available on the other hand.

Clutches and eggs were selected following three “strategies”:

1. Eggs displaying worst cases with regards to several endpoints (eggshell thickness, embryo deformity),
2. Failed eggs of successful clutches with lowest egg hatching rates,
3. Eggs of clutches potentially exposed to specific ASs (ASs that have been commonly used but that would need further consideration with regards to risk assessment for avian reproduction (first-tier toxicity-exposure ratio (TER_lt_) < 5; Bro et al. 2015; EFSA 2009)).

Hatched eggs were not analysed so far both for financial and analytical reasons. Only the calcareous eggshells and chorionic membranes are available. The quantity is likely to be insufficient to allow residue analyses, and this matrix may not be the best one to detect compounds. Additional tests are therefore required.

### Sample size

We performed residue analyses on 52 clutches collected on 12 sites located in intensively cultivated farmlands in north-central France (Bro et al. 2015; Millot et al. 2015). These clutches corresponded to 645 eggs laid, of which 38.8 % hatched. One hundred thirty-nine eggs were analysed, representing 21.6 % of the total number of eggs laid and 35.2 % of failed eggs. The 139 eggs were constituted into 73 samples of one to four eggs. Clutches were analyzed for pesticide residues through one to three samples and one to eight eggs (Table 1).

**Table 1:** Data about clutches and eggs that were analysed for pesticide residues and results of GC/MS-MS and LC/MS-MS analyses (mg/kg)

Clutch code	Clutch characteristics				Egg characteristics				Residue analyses (mg/kg)	
	Fate	Number of eggs laid	% Unhatched eggs^a^	% Fertile eggs	Egg status	Stage of development (days)	Difomity^b^	Eggshell thickness (mm)	GC/MS-MS screening	LC/MS-MS screening
2011-80-864-1	Failure	6	100	42	Dead embryo	3	–	0.236	ND	ND
					Indet	–	–		ND	ND
					Infertile	–	–	0.212		
					Infertile	–	–	0.223		
2011-45-24-2	Hatching	14	86	57	Indet	–	–	0.214	ND	ND
					Indet	–	–	0.254	ND	Thiamethoxam(+clothianidin): *D* = 0.013
					Indet	–	–	0.237		
					Indet	–	–	0.241		
					Indet	–	–	0.226		
					Indet	–	–	0.248	ND	ND
					Indet	–	–	0.251		
					Indet	–	–	0.244		
2011-45-583-2	Hatching	15	67	80	Dead embryo	15	Yes	0.226	ND	ND
					Indet	–	–	0.246	ND	ND
					Indet	–	–	0.27		
					Dead embryo	6	–	0.246		
					Dead embryo	10	–	0.22		
					Dead embryo	11	–	0.418	Heptachlor(+epoxyde) *D* < 0.01	ND
					Indet	–	–	0.206		
					Indet	–	–	0.283		
2011-77J-1153-1	Hatching	10	60	85	Dead embryo	2	–	0.236	ND	ND
					Dead embryo	8	–	0.214	ND	ND
					Indet	–	–	0.212		
					Dead embryo	2	–	0.228		
					Indet	–	–	0.214	ND	ND
					Indet	–	–	0.237		
2011-77P-723-1	Hatching	13	31	96	Dead embryo	22	No	0.198	ND	Bromoxynil *D* < 0.01
					Indet	–	–	0.218	Heptachlor(+epoxyde) *D* = 0.11	ND
					Dead embryo	23	No	0.204		
									Prochloraz(+TCP) *D* = 0.041	
2011-51-104-3	Hatching	11	27	100	Dead embryo	23	No	0.223	ND	ND
					Dead embryo	23	No	0.244	ND	ND
2010-76-359-2	Hatching	11	55	91	Dead embryo^c^	18	Yes	0.186	ND	ND
2010-80-411-2	Hatching	14	93	100	Dead embryo	23	Yes	.	ND	ND
2011-77J-1158-1	Hatching	11	9	100	Dead embryo	22	Yes	0.239	Prochloraz(+TCP) *D* = 0.024	ND
2011-14/61-177-1	Hatching	14	7	93	Infertile	–	–	0.175	ND	ND
2011-41-1101-1	Failure	8	–	–	Dead embryo	2	–	0.127	ND	ND
					Dead embryo	2	–	0.237	DDT(Σ isomers) *D* = 0.046	ND
					Dead embryo	2	–	0.225		
2011-14/61-181-1	Hatching	20	5	100	Dead embryo	9	–	0.239	ND	ND
2011-76-952-1	Hatching	14	21	89	Indet	–	–	0.205	ND	ND
					Indet	–	–	0.25	Fipronil(+sulfone) *D* = 0.0085	ND
					Indet	–	–	0.228	HCH(α + β + δ) *D* = 0.015	
2011-27-1040-1	Hatching	15	20	87	Infertile	–	–	0.205	Cyproconazole *D* = 0.021	ND
2011-28-799-2	Failure	11	100	–	Indet	–	–	0.215	ND	ND
					Indet	–	–	0.277		
					Indet	–	–	0.205		
					Indet	–	–	0.26	ND	ND
					Indet	–	–	0.315		
					Indet	–	–	0.226		
2011-45-586-1	Hatching	18	28	86	Indet	–	–	0.239	ND	ND
					Indet	–	–	0.224		
					Indet	–	–	0.239	Cyhalothrin(lambda) *D* < 0.01	ND
					Indet	–	–	0.258	Fenpropidin *D* = 0.34	
									Heptachlor(+epoxyde) *D* < 0.01	
									Tebuconazole *D* < 0.01	
2011-45-587-1	Hatching	20	30	95	Dead embryo	11	–	0.285	Diphenylamine *D* = 0.01	ND
					Dead embryo	3	–	0.229		
					Dead embryo	12	–	0.251		
					Dead embryo	19	No	0.264	Fenpropidin *D* = 0.036	ND
					Indet	–	–	0.215		
					Indet	–	–	0.25		
2010-78-760-1	Hatching	19	32	95	Dead embryo	12	–	0.245	ND	ND
					Dead embryo	12	–	0.273		
					Dead embryo	12	–	.		
					Dead embryo	12	–	.		
2011-77J-1200-1	Hatching	18	11	94	Indet	–	–	0.223	ND	ND
					Indet	–	–	0.225		
2011-80-871-2	Hatching	13	62	88	Dead embryo	5	–	0.229	Fipronil(+sulfone) *D* = 0.0083	Thiamethoxam(+clothianidin) *D* < 0.01
					Dead embryo	5	–	0.238		
					Indet	–	–	0.214		
					Indet	–	–	0.244	ND	Thiamethoxam(+clothianidin) *D* = 0.037 *of which clothianidin D = 0.032*
					Dead embryo	16	No	0.237	PCB153 *D* < 0.01	–
					Dead embryo	11	–	0.243		
2011-14/61-153-3	Hatching	10	60	85	Infertile	–	–	0.33	ND	ND
					Dead embryo	6	–	0.293		
					Dead embryo	19	No	0.275		
					Indet	–	–	0.351	ND	ND
					Dead embryo	19	No	0.239		
2011-78-51-2	Hatching	11	36	91	Infertile	–	–	0.241	ND	ND
					Dead embryo	23	Yes	0.211	ND	ND
2011-59-848-2	Hatching	4	25	100	Dead embryo	9	–	0.23	ND	ND
2011-51-145-2	Hatching	10	30	85	Infertile	–	–	0.243	Diphenylamine *D* < 0.01	ND
					Dead embryo	18	No	0.219		
2010-51-202-1	Failure	16	–	16	Infertile	–	–	0.253	ND	ND
					Dead embryo	4	–	0.232		
					Dead embryo	3	–	0.265		
					Infertile	–	–	0.235	ND	ND
					Infertile	–	–	0.255		
					Infertile	–	–	0.224		
2011-80-924-1	Failure	15	–	93	Dead embryo	8	–	0.259	ND	ND
					Dead embryo	8	–	0.267		
					Dead embryo	8	–	0.258		
2011-77P-719-1	Hatching	16	25	91	Dead embryo	16	No	0.254	ND	ND
					Indet	–	–	0.231	ND	ND
					Indet	–	–	0.234		
2010-80-442-2	Failure	8	–	100	Dead embryo	23	No	.	ND	ND
2011-41-1101-2	Failure	9	–	78	Infertile	–	–	0.209	DDT(Σ isomers) *D* < 0.01	ND
					Dead embryo	20	No	0.171		
2011-41-1073-1	Hatching	12	33.3	100	Dead embryo	22	Yes	0.223	DDT(Σ isomers) *D* < 0.01	ND
2011-41-1075-1	Failure	7	–	–	Dead embryo	2	–	0.26	DDT(Σ isomers) *D* = 0.027	ND
					Indet	–	–	0.246		
					Dead embryo	2	–	0.25		
2011-41-1107-1	Failure	^3^ 4	–	–	Dead embryo	10	–	0.18	DDT(Σ isomers) *D* = 0.03	ND
					Dead embryo	10	–	0.182		
2011-45-579-1	Hatching	17	11.76	94	Indet	–	–	0.204	Cyproconazole *D* = 0.015	ND
					Indet	–	–	0.226		
2011-41-1066-1	Failure	22	–	100	Dead embryo	15	No		ND	ND
2011-45-658-1	Failure	15	–	100	Dead embryo	20	No	0.238	DDT(Σ isomers) *D* = 0.016	ND
2011-59-841-1	Failure	15	–	67	Dead embryo	2	–	0.218	Fipronil(+sulfone) *D* = 0.0068	Thiamethoxam(+clothianidin) *D* = 0.067 *of which clothianidin D = 0.057*
									PCB153 *D* < 0.01	
					Dead embryo	2	–	0.234	PCB180 *D* < 0.01	
					Infertile	–	–	0.212		
2011-59-803-1	Failure	12	–	83	Dead embryo	4	–	0.25	Prochloraz(+TCP) *D* < 0.01	ND
					Dead embryo	5	–	0.239		
					Dead embryo	4	–	0.253		
2011-59-839-2	Hatching	8	12.5	94	Indet	–	–	0.252	ND	ND
2011-80-906-1	Failure	15	–	93	Indet	–	–	0.25	Prochloraz(+TCP) *D* < 0.01	ND
					Indet	–	–	0.245		
					Dead embryo	21	No	0.21	Prochloraz(+TCP) *D* < 0.01	ND
2011-80-886-1	Failure	16	–	97	Indet	–	–	0.215	Diflufenican *D* = 0.016	ND
					Dead embryo	22	No	0.216		
2011-80-917-1	Failure	14	–	100	Dead embryo	21	No	0.234	PCB153 *D* < 0.01	ND
2011-14-61-168-2	Failure	14	–	96	Dead embryo	20	No	0.242	ND	ND
					Indet	–	–	0.22		
2011-14-61-175-1	Failure	8	–	–	Dead embryo	10	–	0.236	ND	ND
2011-51-111-1	Failure	14	–	7	Infertile	–	–	0.231	Fenpropodin *D* = 0.032	ND
					Dead embryo	2	–	0.22		
2011-51-204-1	Failure	10	–	80	Dead embryo	18	No	0.225	ND	ND
					Dead embryo	18	No	0.221		
					Dead embryo	18	No	0.24		
2011-51-222-2	Failure	32	–	–	Dead embryo	12	–	0.182	ND	ND
					Infertile	–	–	0.165		
2011-77J-1148-1	Hatching	18	11.1	94	Indet	–	–	0.243	ND	ND
					Indet	–	–	0.268		
2011-77P-1164-1	Hatching	14	21.4	93	Dead embryo	10	–	0.209	ND	ND
					Dead embryo	22	Yes	0.238		
2011-76-359-1	Failure	13	–	31	Dead embryo	2	–	0.243	ND	ND
					Infertile	–	–	0.23		
					Dead embryo	2	–	0.251		
2011-27-38-1	Failure	12	–	67	Dead embryo	21	Yes	0.221	ND	ND
2011-76-999-1	Failure	31	–	–	Dead embryo	2	–	0.24	ND	ND
2011-76-958-2	Failure	8	–	63	Dead embryo	20	No	0.238	Difenoconazole *D* = 0.013	ND
									Diphenylamine *D* = 0.019	

*ND* no active substances (ASs) detected (LoD approximatively half of LoQ—0.01 mg/kg for most ASs, see Online Resource 1)), *–* not relevant data

^a^Calculated on clutches incubated ≥24 days

^b^Recorded for embryo ≥15 days of development

^c^Only the embryo was available for residue analysis

### Potential exposure

We considered that a female (and then her eggs) was potentially exposed to an AS if the area where it was radiotracked during the pre-laying, egg-laying or incubation periods overlapped with treated fields (see Bro et al. 2015 for methodological details). When a compound was detected in a clutch, we provided data (date, crop, dose of AS(s)) about the corresponding treatment(s) of the field(s) frequented by the females and their eggs. We calculated the amount of the AS applied on the field (g/ha) by multiplying the dose (l/ha) applied by the farmers and the concentration of the AS in the trade formulation (mg/l or g/l). The former was known from a farmer survey^2^ (Bro et al. 2015), the latter from information retrieval in the E-PHY database managed by the French Ministry of Agriculture (http://e-phy.agriculture.gouv.fr/).

## Results

### Clutch contamination

We detected contaminants in 24 out of the 52 analysed clutches, i.e., 46.2 % (Table 1). Globally, few compounds were detected compared to what one could have expected on the basis of potential exposure (Online Resources 2 and 3). A total of 15 different compounds were found, of which nine ASs are currently used by farmers to protect crops. The detected substances were azole (difenoconazole, tebuconazole, cyproconazole, prochloraz) or amine (fenpropidin) fungicides, pyrethroid and neonicotinoid insecticides (lambda-cyhalothrin and thiamethoxam/clothianidin, respectively) and herbicides (bromoxynil—hydroxybenzonitrile and diflufenican—pyridinecarboxamide). The other compounds were fipronil(+sulfone), HCH(α,β,δ isomers), diphenylamine, heptachlor(+epoxyde), DDT(Σisomers) and PCBs(153, 180).

We detected one compound in 70.8 % of the 24 contaminated clutches, two compounds in 12.5 % and more complex mixtures (three to five compounds) in 16.7 %. In one clutch, we detected two compounds that were combined in a trade co-formulation (tebuconazole and fenpropidin; cf. Online Resource 3: clutch 2011-45-586-1).

### Concentrations

A compound was detected but not quantified in 14 cases (Table 1). When concentrations were quantified, they were generally within one to four/five orders of magnitude of the LoQ. Higher concentrations were quantified in three cases: 0.067 mg/kg of thiamethoxam/clothianidin, 0.11 mg/kg of heptachlor(+epoxyde) and 0.34 mg/kg of fenpropidin (Table 1).

### Contamination and exposure

In eight cases, we could associate the contamination of the clutch to a potential exposure of the female during egg formation and/or egg laying (Online Resource 3). The fungicide fenpropidin was frequently detected when “expected” (three clutches out of four potentially exposed, Online Resource 2), but this was not observed for the other ASs. The dose applied on the field often corresponded to a low volume compared to the approved maximum dose.

In nine other cases, contamination could not be associated with a potential exposure of the female identified using the method of Bro et al. (2015), but the AS has been used in the study site of the corresponding year. Data are provided in the last column (“other uses in the site”) in Online Resource 3. In other cases, the compounds that were detected were not listed as ASs used by the farmers (and not as a pesticide in case of PCBs).

## Discussion

### Contamination of eggs by current active substances

Contamination of farmland birds by the PPPs currently used in agriculture is poorly documented in situ, with some exceptions such as raptor species (see Gómez-Ramírez et al. 2014). In this context, this study provides some field data on a typical medium-sized omnivorous farmland bird, the grey partridge. It reports the contamination of some eggs by some current ASs. Such result is a first stone in the foundation of further investigations of unintentional effects of PPPs on non-target wildlife in cultivated landscapes. The second main contribution of this work is to provide field references of concentration of some ASs in eggs, and data about the treatments of the field(s) frequented by the females and/or where their eggs were laid. However, it remains difficult in the state of the art to relate concentrations in eggs to exposure doses given the gaps of knowledge (identification and quantification of the different routes of exposure) and of data (transfer characteristics in eggs) in birds. In spite of this, our results would be useful to plan lab experiments with realistic in ovo injections (e.g., Blus and Henny 1997; Dunachie and Fletcher 1970; Fischer 2005).

We detected nine ASs out of the hundred to which eggs have been potentially exposed to (Bro et al. 2015, Online Resource 2). It would have been be quite interesting to correlate the detection of ASs to a series of factors such as their physico-chemical properties, their environmental fate, their metabolism in birds (see data compiled in Online Resource 4), as well as the associated treatments (crop, usage, time and type of application, dose applied, window of exposure, etc.; see data in Online Resource 3). Unfortunately, available data of contamination are not sufficient so far to provide a reliable analysis. In particular, absence of detection and detection of mixtures should be consolidated by a higher sample size.

Contamination by thiamethoxam and clothianidin should be discussed with regards to the current regulatory ban of some neonicotinoids in some countries. These ASs were detected as thiamethoxam/clothianidin in three clutches laid in 2011 in three different geographical sites (Online Resource 3). This contamination has probably been detected as a result of exposure to thiamethoxam. Indeed, it was used on these three sites between March and May, when sowing beet, maize or pea seeds—authorised uses in 2011 (Online Resource 3). Two females frequented one or two fields of maize in April and one female a field of canning peas in May. Thus, exposure is therefore highly credible knowing the diet and the habitat use of the species. These potential exposures were not identified by Bro et al. (2015) because they considered a 15-day period before the laying date of the first egg (this corresponds to the main phase of yolk formation in the domestic hen) to determine potential exposure. Sowing of coated seeds occurred before that time.

Clothianidin was identified as the main residue in two samples (Online Resource 3). This is compatible with an exposure to thiamethoxam since clothianidin is a metabolite of thiamethoxam metabolism in the domestic hen (EFSA 2014). Clothianidin is maybe a residue also found in eggs (this is not mentioned in the report) given that it is the major residue found in the liver. More quantitative data would be welcome to interpret more deeply our results. However, we cannot exclude an exposure to clothianidin given that this AS was authorised in France on maize from 1 April to 31 July 2011, as in-furrow application of pellets (ANSES 2011; DRIAAF 2011). Parent compound is reported to account for 20 % of the radioactivity of residues in eggs following an exposure of laying hens to clothianidin (EFSA 2014). This scenario is however not the most probable according to our field data.

#### Routes of contamination

Four routes of contamination were possible: (1) a direct exposure of the eggs when the pesticides were sprayed, (2) a direct exposure of the eggs through the contaminated vegetation, (3) a direct exposure of the eggs through the contaminated feathers of the females and (4) a maternal transfer to the eggs (through diet, preening and/or inhalation). They are not mutually exclusive. All were considered in the method that was proposed by Bro et al. (2015) to identify and quantify potential exposure.

Direct exposure is a likely phenomenon both because most ASs applied in the fields during the breeding season are sprayed (Millot et al. 2015) and because the grey partridge is a terrestrial bird that mainly lives and nests in crops (Bro et al. 2013). However, whether direct exposure is an important route of egg contamination is an open question. The behaviour of grey partridge females may prevent direct exposure from spraying as well as favour it from treated vegetation, depending upon the relative dates of treatments and laying. Indeed, laying females only remain on their nests during a few hours per day to lay (Birkan and Jacob 1988), and they cover their eggs with plant material found around the nest when they leave it (McCabe and Hawkins 1946). A direct exposure of the clutch when the crop where it was laid has been treated may have occurred in four cases out of the eight contaminated clutches with a potential exposure identified with the method used by Bro et al. (2015).

Maternal exposure is likely to be an important route of exposure given (1) the timing of pesticide use (mainly during egg formation, Bro et al. 2015), (2) the diet of the species and (3) the biochemical origin of egg content (lipovitellin and phosvitin, some yolk lipoproteins, are exclusively synthetised by the liver of the laying hen (Sauveur and de Reviers 1988); thus, lipophilic ASs may be included in fatty content). In addition, the metabolism studies performed in laying hens indeed demonstrate a residue transfer to the eggs for many ASs (Online Resource 4, EFSA reasoned opinions). Exposure through inhalation cannot be excluded given that some of the ASs are volatile (Online Resource 4) and are detected in the air (Marlière 2009).

#### Which level of contamination at the population scale?

We detected compounds in slightly less than half of the clutches we analysed, and the ASs were generally quantified at low concentrations, i.e., close to the limit of quantification. One of the two issues now is to know whether it rather reflects a low or a high level of contamination at the population scale. At the current stage of the work, it is difficult to draw a conclusion. Indeed, egg sample is biased toward unhatched eggs. This may overestimate the detection of contaminations if contaminations reduce egg hatching rate, as well as underestimate it if contaminations only impact hatchling conditions (e.g., Kitulagodage et al. 2011). Laying order may also have influenced the probability to detect an exposure to an AS, but this aspect is not known with our field data.

The probability to detect contaminations in situ taking into account the different mechanisms of losses and transfer should also be considered to interpret the results. We did not find data about transfer of ASs into egg content through eggshell and membranes for the ASs under interest in case of a direct exposure despite immersion experiments have been performed (Dunachie and Fletcher 1966, 1967). Transfer rate is increased when a fat-soluble AS is associated to oil vehicle (Hoffman and Albers 1984). If egg contamination results from a maternal effect, we need to look at the metabolism of the AS in birds. Detailed data exist on poultry. Experiments on laying hens administered (chronically or not) with radiolabelled ASs provide information on the excretion rate and on the kinetics of the ASs, as well as on the distribution of residues in organs, tissues and eggs. Partial data can be found in public reports and databases (Online Resource 4). Given that domestic hens and grey partridges are both galliform species, we assume that hen data are representative of our species. Data on metabolism in laying hens indicate that the (at least some) ASs are rapidly and mainly excreted via the urine and/or the faeces. Only small amounts of residues are transferred to the eggs. In the light of these pieces of information, we expect to only detect a maternal contamination as a “weak signal” given the probability to capture such event. This issue has been already highlighted as inherent to field conditions (Quintaine et al. in press), and abnormality frequency may be more informative than deviations from means (Egea-Serrano et al. 2012). Under this hypothesis, and taking into account any prior degradation in the environment (metabolism in plants, phytolysis, etc.), our results might indicate a more global non-target pesticide exposure of farmland birds.

#### Effects on breeding performance

The second key issue is to relate residues in eggs to effects on the individuals (health concern) and *in fine* on the population dynamics (population management concern). Correlating actual exposure (presence and concentration of ASs) to the characteristics of individual eggs (fate, eggshell thickness) and embryos (stage of development when death occurred, deformity) would have required to perform a separate analysis for each egg and to analyse simultaneously control eggs. Other endpoints such as the oxidative stress may also be valuable additive information.

Some ASs detected in partridge eggs may influence, under certain exposure conditions, the fecundity, the fertility and the development of embryos of some bird species (Lopez-Antia et al. 2013; Rivière et al. 1985) or are suspected to have endocrine disrupting properties (e.g., EFSA 2010, 2011; Saxena et al. 2015). However, whether such effects actually occur in the field remains an open question. This issue is still a current challenge in avian ecotoxicology. It is all the more complex to be documented that a series of other causes may contribute to variations in clutch size (Mourão et al. 2010), fertility rate (Bramwell 2002) and embryonic mortality rate (Mourão et al. 2010; Nakage et al. 2003; Wilson et al. 2003). In this context, field (Bishop et al. 2000), semi-field (Johnston et al. 1996) and/or lab (Lopez-Antia et al. 2013, 2015; Kitulagodage et al. 2011) experimental studies are required to control potential confounding factors and provide conclusive results.

### Contaminations by other pollutants

We detected in partridge eggs some compounds (DDT/isomers, PCB/congeners, heptachlor, HCH/isomers) that are listed on annex A or B of the Stockholm convention on persistent organic pollutants (POPs). These chemicals are widely distributed and persistent in the environment, they bioaccumulate through the food web and are toxic to humans and wildlife. As such, they still receive special attention from researchers. As a consequence and contrarily to current pesticide ASs, a large body of literature is devoted to these compounds in wild birds (e.g., Fernie et al. 2003; Fry 1995; Gómez-Ramírez et al. 2014; Hoffman et al. 2002; Meador 1996). These compounds are still detected in many bird species throughout the world (e.g., Augspurger et al. 2008—wood duck in USA; Clark et al. 2009—peregrine falcon in USA; Eng et al. 2014—starling in Canada; Gomez-Ramirez et al. 2012; Martínez-López et al. 2007—Eurasian eagle owl, booted eagle and goshawk in Spain; Fliedner et al. 2012—herring gull in Germany; Gao et al. 2009—six species of aquatic birds (gull, tern, plover, common coot), ring-necked pheasant, mallard and swan in China; Kocagöz et al. 2014—gulls, coot and heron in Turkey; Malik et al. 2011—cattle egret in Pakistan). Our results provide however new data since little is known about their occurrence in free-living galliform birds. The concentrations are however quite lower than the ones currently quantified in other species/countries and are not likely to reduce the breeding success of the grey partridge.

Soil particles ingested with grit or soil residues absorbed by plants may be the exposure route for compounds such as HCH (Orton et al. 2013), fipronil or neonicotinoids (Bonmatin et al. 2015) that are stored in French soils. The spot of contamination by DDT/isomers is less obvious, and only hypotheses can be proposed. In some raptor studies, it is suggested that the recent increase of DDE concentrations in eggs are due to a local use of dicofol (*in* Gómez-Ramírez et al. 2014; Wiemeyer et al. 1989, 2001), an organochlorine acaricide manufactured from DDT used to protect vegetables and fruits (JMPR – Joint FAO/WHO Meeting on Pesticide Residues 2011). International regulations limit impurities in technical dicofol, but accidents in pesticide refinement are possible. Such an origin in our study may be plausible given that (i) DDT/isomers were detected in clutches of partridges living in a site where strawberries, legumes and wine are produced and (ii) its use as an acaricide on strawberries, beans and grapes was banned in France in late March 2010 (EPHY database, accessed September 2015). Although this AS was not listed in the course of our study, we cannot exclude that it has been used by some farmers that did not participate to the work (see Bro et al. 2015; Millot et al. 2015).

## Conclusion

This work provides evidence of the contamination of a farmland galliform bird by some pesticide ASs. The characteristics of rapid and high excretion rate of ASs and their low transfer rate to tissues, organs and eggs measured in worst-case situations do not fully prevent contamination of non-target organisms in the real world. This result is congruent with risk assessment estimates of some ASs (higher tier TER lower or close to 5, EFSA 2009). However, the effects of the contamination we detected on egg characteristics and egg fate cannot be inferred from our data. It is known from lab studies that exposure to some ASs, of which chemicals suspected to have endocrine disruptor properties, during embryonic development may have reproductive consequences, but whether such effects occur in the field remains an open question. Conclusions will only be drawn from experiments simulating operational conditions of use. However, before planning experiments, exposure in field conditions should be better documented. Provided that funds were available, partridge failed eggs might be a non-invasive monitoring tool of contamination of farmland birds by PPPs. Indeed, hunters are solicited to collect clutches deserted by partridges following disturbance, for instance by crop harvesting, and to make the eggs incubated by bantam hens or artificially in specific centres. A proportion of eggs could be analysed. Such dedicated surveys are already operational for monitoring wildlife poisoning incidents (e.g., SAGIR in France—http://www.oncfs.gouv.fr/Reseau-SAGIR-ru105, the Wildlife Incident Investigation Scheme in UK—http://www.pesticides.gov.uk/guidance/industries/pesticides/topics/reducing-environmental-impact/wildlife) or monitoring some contaminants in some raptor species and their eggs (reviewed by Gómez-Ramírez et al. 2014). Such a wide-scale scheme could be implemented for farmland birds and PPPs. It would be highly valuable as a surveillance tool of pesticide contamination of wildlife as a complement of more focused studies (Quintaine et al. in press; Lopez-Antia et al. 2013, 2015).

## Electronic supplementary material

Below is the link to the electronic supplementary material.

Online Resource 1 Online Resource 1 Compounds measured in the GC/MS-MS and LC/MS-MS multi residue analyses, and associated limit of quantification (LoQ). References – GC: FB3/02.c vers. 8 (30/01/2015); LC: FB3/02.e vers. 9 (30/01/2015). Compounds are arranged alphabetically (PDF 26 kb)

Online Resource 2 Online Resource 2 Active substances (ASs, *n* = 179) listed as used in intensively cultivated farmlands in north-central France in spring and summer 2010–2011, statistics of use, potential exposure, and number of clutches where the AS was detected. Figures into brackets refer to the number of clutches where the AS was detected with no potential exposure of the clutch identified with the method used by Bro et al. (2015). The other compounds detected in the clutches are also provided in the list. Compounds are arranged alphabetically (PDF 13 kb)

Online Resource 3 Online Resource 3 Compounds detected in the 24 clutches out of the 52 clutches analysed for pesticide residues, and treatments that are likely to have caused the exposure (uses in 2010–2011). Lines separate the different clutches (PDF 18 kb)

Online Resource 4 Online Resource 4 Information about compounds that were detected in partridge’ eggs. The dash “−” indicates that the information was not found. (PDF 41.4 kb)

## Footnotes

1 See Online Resource 4.

2 Field data “checked” on E-PHY
