# Supplementary material for: Residues of plant protection products in grey partridge eggs in French cereal ecosystems
Source: Environ Sci Pollut Res Int. 2016 Feb 3;23:9559–73. doi: 10.1007/s11356-016-6093-7 (PMC4871908; doi:10.1007/s11356-016-6093-7)
Supplement: Supplementary file 1 — Online Resource 1 Compounds measured in the GC/MS-MS and LC/MS-MS multi residue analyses, and associated limit of quantification (LoQ). References – GC: FB3/02.c vers. 8 (30/01/2015); LC: FB3/02.e vers. 9 (30/01/2015). Compounds are arranged alphabetically (PDF 26 kb) [file 11356_2016_6093_MOESM1_ESM.pdf]

| Compound | Method | LoQ (mg/kg) |
|---|---|---|
| 2,4,5-T | LC/MS-MS | 0.01 |
| 2,4D (free acid) | LC/MS-MS | 0.01 |
| 2,4DB | LC/MS-MS | 0.01 |
| 2-Phenylphenol | GC/MS-MS | 0.01 |
| 3,4-dichloroaniline | GC/MS-MS | 0.01 |
| 6-Benzyladenine | LC/MS-MS | 0.01 |
| Abamectine(∑B1a+B1b) | LC/MS-MS | 0.01 |
| Acephate | GC/MS-MS | 0.01 |
| Acequinocyl | LC/MS-MS | 0.01 |
| Acetamipride | LC/MS-MS | 0.01 |
| Acetochlore | GC/MS-MS | 0.01 |
| Acibenzolar-S-methyl | GC/MS-MS | 0.01 |
| Aclonifen | GC/MS-MS | 0.01 |
| Acrinathrine | GC/MS-MS | 0.01 |
| Alachlore | GC/MS-MS | 0.01 |
| Aldicarb(+sulfoxide) | LC/MS-MS | 0.01 |
| Ametoctradine | LC/MS-MS | 0.01 |
| Ametryn | GC/MS-MS | 0.01 |
| Amidosulfuron | LC/MS-MS | 0.01 |
| Amitraze(+2,4 dimethylaniline) | LC/MS-MS | 0.01 |
| Atrazine | GC/MS-MS | 0.01 |
| Atrazine-desethyl(+deisopropyl) | LC/MS-MS | 0.01 |
| Azaconazole | LC/MS-MS | 0.01 |
| Azimsulfuron | LC/MS-MS | 0.01 |
| Azinphos-ethyl | LC/MS-MS | 0.01 |
| Azinphos-methyl | LC/MS-MS | 0.01 |
| Azoxystrobine | LC/MS-MS | 0.01 |
| Beflubutamide | LC/MS-MS | 0.01 |
| Benalaxyl (+Benalaxyl-M) | GC/MS-MS | 0.01 |
| Bendiocarb | GC/MS-MS | 0.01 |
| Benfluraline | GC/MS-MS | 0.01 |
| Benfuracarbe | LC/MS-MS | 0.01 |
| Benoxacor | GC/MS-MS | 0.01 |
| Bensulfuron-methyl | LC/MS-MS | 0.01 |
| Bentazone(+Bentazone 8-OH) | LC/MS-MS | 0.01 |
| Benthiavalicarb-isopropyl | LC/MS-MS | 0.01 |
| Bifenazate | LC/MS-MS | 0.01 |
| Bifenox | GC/MS-MS | 0.01 |
| Bifenthrine | GC/MS-MS | 0.01 |
| Biphenyl | GC/MS-MS | 0.01 |
| Bispyribac-sodium | LC/MS-MS | 0.01 |
| Bitertanol | GC/MS-MS | 0.01 |
| Bixafen | LC/MS-MS | 0.01 |
| Boscalide | LC/MS-MS | 0.01 |
| Bromacil | GC/MS-MS | 0.01 |
| Bromocyclen | GC/MS-MS | 0.01 |
| Bromophos-ethyl | GC/MS-MS | 0.01 |
| Bromophos-methyl | GC/MS-MS | 0.01 |
| Bromopropylate | GC/MS-MS | 0.01 |
| Bromoxynil | LC/MS-MS | 0.01 |
| Bromuconazole | LC/MS-MS | 0.01 |
| Bupirimate | LC/MS-MS | 0.01 |
| Buprofezin | LC/MS-MS | 0.01 |
| Butachlor | GC/MS-MS | 0.01 |
| Butafenacil | LC/MS-MS | 0.01 |
| Butraline | GC/MS-MS | 0.01 |
| Buturon | LC/MS-MS | 0.01 |
| Cadusafos | LC/MS-MS | 0.01 |
| Captafol | GC/MS-MS | 0.01 |
| Captan | GC/MS-MS | 0.01 |
| Carbaryl | GC/MS-MS | 0.01 |
| Carbendazime(+Benomyl) | LC/MS-MS | 0.01 |
| Carbetamide | LC/MS-MS | 0.01 |
| Carbofuran(+3-hydroxy) | GC/MS-MS | 0.01 |
| Carbophenothion | GC/MS-MS | 0.01 |

| Compound | Method | LoQ (mg/kg) |
|---|---|---|
| Carbosulfan | LC/MS-MS | 0.01 |
| Carboxine | LC/MS-MS | 0.01 |
| Carfentrazone-ethyl | GC/MS-MS | 0.01 |
| Chlorantraniliprole | LC/MS-MS | 0.01 |
| Chlorbenside | GC/MS-MS | 0.01 |
| Chlordane(cis+trans) | GC/MS-MS | 0.01 |
| Chlorfenapyr | GC/MS-MS | 0.01 |
| Chlorfenson | GC/MS-MS | 0.01 |
| Chlorfenvinphos | GC/MS-MS | 0.01 |
| Chloridazon | LC/MS-MS | 0.01 |
| Chlorobenzilate | GC/MS-MS | 0.01 |
| Chlorothalonil | GC/MS-MS | 0.01 |
| Chlorotoluron | LC/MS-MS | 0.01 |
| Chloroxuron | LC/MS-MS | 0.01 |
| Chloroxynil | LC/MS-MS | 0.01 |
| Chlorprophame(+3-Chloroaniline) | GC/MS-MS | 0.01 |
| Chlorpyrifos | GC/MS-MS | 0.01 |
| Chlorpyrifos-methyl | GC/MS-MS | 0.01 |
| Chlorsulfuron | LC/MS-MS | 0.01 |
| Chlorthal-dimethyl | GC/MS-MS | 0.01 |
| Chlorthiophos | GC/MS-MS | 0.01 |
| Chlozolinate | GC/MS-MS | 0.01 |
| Chromafenozide | LC/MS-MS | 0.01 |
| Cinidon-ethyl | LC/MS-MS | 0.01 |
| Cinosulfuron | LC/MS-MS | 0.01 |
| Clethodim+Sethoxydim | LC/MS-MS | 0.01 |
| Clodinafop(∑isomers) | LC/MS-MS | 0.01 |
| Clodinafop-propargyl | GC/MS-MS | 0.01 |
| Clofentezine | LC/MS-MS | 0.01 |
| Clomazone | GC/MS-MS | 0.01 |
| Cloquintocet-mexyl | LC/MS-MS | 0.01 |
| Clothianidine | LC/MS-MS | 0.01 |
| Coumaphos | GC/MS-MS | 0.01 |
| Cyanazine | LC/MS-MS | 0.01 |
| Cyazofamide | LC/MS-MS | 0.01 |
| Cycloxydime | LC/MS-MS | 0.01 |
| Cycluron | LC/MS-MS | 0.01 |
| Cyflufenamid | LC/MS-MS | 0.01 |
| Cyfluthrine(β+γ) | GC/MS-MS | 0.01 |
| Cyhalofop-butyl | GC/MS-MS | 0.01 |
| Cymiazole | GC/MS-MS | 0.01 |
| Cymoxanil | LC/MS-MS | 0.01 |
| Cypermethrine(α+β+θ+ζ) | GC/MS-MS | 0.01 |
| Cyproconazole | GC/MS-MS | 0.01 |
| Cyprodinil | GC/MS-MS | 0.01 |
| Cyromazine | LC/MS-MS | 0.01 |
| Dazomet | LC/MS-MS | 0.01 |
| DDT(Σisomers) | GC/MS-MS | 0.01 |
| Deltamethrine | GC/MS-MS | 0.01 |
| Demeton-S | LC/MS-MS | 0.01 |
| Demeton-S-methyl(sulfone+sulfon) | LC/MS-MS | 0.01 |
| Demeton-S-methyl | GC/MS-MS | 0.01 |
| Desmediphame | LC/MS-MS | 0.01 |
| Desmetryn | LC/MS-MS | 0.01 |
| Diafenthiuron | LC/MS-MS | 0.01 |
| Dialifos | GC/MS-MS | 0.01 |
| Diallate | GC/MS-MS | 0.01 |
| Diazinon | GC/MS-MS | 0.01 |
| Dicamba | LC/MS-MS | 0.01 |
| Dichlobenil | GC/MS-MS | 0.01 |
| Dichlofenthion | GC/MS-MS | 0.01 |
| Dichlofluanide | GC/MS-MS | 0.01 |
| Dichlorprop(free acid) | LC/MS-MS | 0.01 |
| Dichlorvos | GC/MS-MS | 0.01 |
| Diclobutrazol | LC/MS-MS | 0.01 |

| Compound | Method | LoQ (mg/kg) |
|---|---|---|
| Diclofop(free acid) | LC/MS-MS | 0.01 |
| Diclofop-methyl | GC/MS-MS | 0.01 |
| Dicloran | LC/MS-MS | 0.01 |
| Dicofol(Σisomers) | GC/MS-MS | 0.01 |
| Dieldrin(+Aldrin) | GC/MS-MS | 0.01 |
| Diethofencarb | GC/MS-MS | 0.01 |
| Difenacoum | LC/MS-MS | 0.01 |
| Difenamide | LC/MS-MS | 0.01 |
| Difenoconazole | GC/MS-MS | 0.01 |
| Difethialone | LC/MS-MS | 0.01 |
| Diflubenzuron | LC/MS-MS | 0.01 |
| Diflufenican | GC/MS-MS | 0.01 |
| Dimetachlor | GC/MS-MS | 0.01 |
| Dimethenamid-P(Σisomers) | LC/MS-MS | 0.01 |
| Dimethoate(+Omethoate) | LC/MS-MS | 0.01 |
| Dimethomorphe(Σisomers) | LC/MS-MS | 0.01 |
| Dimoxystrobine | LC/MS-MS | 0.01 |
| Diniconazole(Σisomers) | LC/MS-MS | 0.01 |
| Dinitramine | GC/MS-MS | 0.01 |
| Dinocap(Σisomers) | LC/MS-MS | 0.01 |
| Dinoseb | LC/MS-MS | 0.01 |
| Dinotefuran | LC/MS-MS | 0.01 |
| Dinoterb | LC/MS-MS | 0.01 |
| Dioxathion | LC/MS-MS | 0.01 |
| Diphenylamine | GC/MS-MS | 0.01 |
| Disulfoton | GC/MS-MS | 0.01 |
| Disulfoton-sulfoxe(+sulfoxide) | LC/MS-MS | 0.01 |
| Ditalimfos | GC/MS-MS | 0.01 |
| Dithianon | LC/MS-MS | 0.01 |
| Diuron | LC/MS-MS | 0.01 |
| DMST | LC/MS-MS | 0.01 |
| DNOC | LC/MS-MS | 0.01 |
| Dodemorphe | LC/MS-MS | 0.01 |
| Dodine | LC/MS-MS | 0.01 |
| Edifenphos | GC/MS-MS | 0.01 |
| Emamectine-benzoate B1b | LC/MS-MS | 0.01 |
| Emamectine-benzoate B1a | LC/MS-MS | 0.01 |
| Endosulfan(α+β+sulfate) | GC/MS-MS | 0.01 |
| Endrin | GC/MS-MS | 0.01 |
| Endrin-ketone | GC/MS-MS | 0.01 |
| EPN | GC/MS-MS | 0.01 |
| Epoxiconazole | LC/MS-MS | 0.01 |
| EPTC | GC/MS-MS | 0.01 |
| Ethalfluraline | GC/MS-MS | 0.01 |
| Ethidimuron | LC/MS-MS | 0.01 |
| Ethiofencarb | GC/MS-MS | 0.01 |
| Ethiofencarb-sulfone(+sulfoxide) | LC/MS-MS | 0.01 |
| Ethion | GC/MS-MS | 0.01 |
| Ethofumesate | GC/MS-MS | 0.01 |
| Ethoprophos | GC/MS-MS | 0.01 |
| Ethoxyquine | GC/MS-MS | 0.01 |
| Etofenprox | GC/MS-MS | 0.01 |
| Etoxazole | LC/MS-MS | 0.01 |
| Etridiazole | GC/MS-MS | 0.01 |
| Etrimfos | GC/MS-MS | 0.01 |
| Famoxadone | GC/MS-MS | 0.01 |
| Famphur | GC/MS-MS | 0.01 |
| Fenamidone | LC/MS-MS | 0.01 |
| Fenamiphos | GC/MS-MS | 0.01 |
| Fenamiphos-sulfone(+sulfoxide) | LC/MS-MS | 0.01 |
| Fenarimol | GC/MS-MS | 0.01 |
| Fenazaquin | GC/MS-MS | 0.01 |
| Fenbuconazole | LC/MS-MS | 0.01 |
| Fenchlorphos(+oxon) | GC/MS-MS | 0.01 |
| Fenhexamide | GC/MS-MS | 0.01 |

| Compound | Method | LoQ (mg/kg) |
|---|---|---|
| Fenitrothion | GC/MS-MS | 0.01 |
| Fenobucarbe | GC/MS-MS | 0.01 |
| Fenoxaprop-ethyl | GC/MS-MS | 0.01 |
| Fenoxycarbe | GC/MS-MS | 0.01 |
| Fenpropathrine | GC/MS-MS | 0.01 |
| Fenpropidine | GC/MS-MS | 0.01 |
| Fenpropimorphe | GC/MS-MS | 0.01 |
| Fenpyroximate | LC/MS-MS | 0.01 |
| Fensulfothion(+sulfone) | GC/MS-MS | 0.01 |
| Fensulfothion-oxon(+sulfone) | LC/MS-MS | 0.01 |
| Fenthion(+sulfone+sulfoxide) | GC/MS-MS | 0.01 |
| Fenthion-oxon(+sulfone+sulfoxide) | LC/MS-MS | 0.01 |
| Fenuron | LC/MS-MS | 0.01 |
| Fenvalerate(Σisomers) | GC/MS-MS | 0.01 |
| Fipronil(+sulfone) | GC/MS-MS | 0.005 |
| Fipronil-desulfinyl | GC/MS-MS | 0.01 |
| Flazasulfuron | LC/MS-MS | 0.01 |
| Flonicamide | LC/MS-MS | 0.01 |
| Flonicamide(+TNFA+TNFG) | LC/MS-MS | 0.05 |
| Florasulam | LC/MS-MS | 0.01 |
| Fluazifop(free acid) | LC/MS-MS | 0.01 |
| Fluazifop-p-butyl | GC/MS-MS | 0.01 |
| Fluazinam | LC/MS-MS | 0.01 |
| Flubendiamide | LC/MS-MS | 0.01 |
| Fluchloralin | GC/MS-MS | 0.01 |
| Flucythrinate | GC/MS-MS | 0.01 |
| Fludioxonil | GC/MS-MS | 0.01 |
| Flufenacet | GC/MS-MS | 0.01 |
| Flufenoxuron | LC/MS-MS | 0.01 |
| Fluometuron | LC/MS-MS | 0.01 |
| Fluopicolide | GC/MS-MS | 0.01 |
| Fluopyram | LC/MS-MS | 0.01 |
| Fluoxastrobine | LC/MS-MS | 0.01 |
| Fluquinconazole | LC/MS-MS | 0.01 |
| Flurochloridone | GC/MS-MS | 0.01 |
| Fluroxypyr(free acid) | LC/MS-MS | 0.01 |
| Fluroxypyr-methylheptyl ester | GC/MS-MS | 0.01 |
| Flurtamone | LC/MS-MS | 0.01 |
| Flusilazole | GC/MS-MS | 0.01 |
| Fluthiacet-methyl | LC/MS-MS | 0.01 |
| Flutolanil | GC/MS-MS | 0.01 |
| Flutriafol | GC/MS-MS | 0.01 |
| Fluvalinate(Tau) | GC/MS-MS | 0.01 |
| Fluxapyroxad | LC/MS-MS | 0.01 |
| Folpet | GC/MS-MS | 0.01 |
| Fomesafen | LC/MS-MS | 0.01 |
| Fonofos | GC/MS-MS | 0.01 |
| Foramsulfuron | LC/MS-MS | 0.01 |
| Forchlorfenuron | LC/MS-MS | 0.01 |
| Formetanate(hydrochlorure) | LC/MS-MS | 0.01 |
| Formothion | GC/MS-MS | 0.01 |
| Fosthiazate | LC/MS-MS | 0.01 |
| Fuberidazole | LC/MS-MS | 0.01 |
| Furalaxyl | GC/MS-MS | 0.01 |
| Furathiocarbe | GC/MS-MS | 0.01 |
| Furmecyclox | LC/MS-MS | 0.01 |
| Halosulfuron-methyl | LC/MS-MS | 0.01 |
| Haloxyfop(free acid) | LC/MS-MS | 0.01 |
| Haloxyfop-2-ethoxyethyl | GC/MS-MS | 0.01 |
| Haloxyfop-methyl(R+S) | GC/MS-MS | 0.01 |
| HCB | GC/MS-MS | 0.01 |
| HCH(γ) | GC/MS-MS | 0.01 |
| HCH(α+β+δ) | GC/MS-MS | 0.01 |
| Heptachlore(+epoxyde) | GC/MS-MS | 0.01 |
| Heptenophos | GC/MS-MS | 0.01 |

| Compound | Method | LoQ (mg/kg) |
|---|---|---|
| Hexaconazole | GC/MS-MS | 0.01 |
| Hexaflumuron | LC/MS-MS | 0.01 |
| Hexazinone | GC/MS-MS | 0.01 |
| Hexythiazox | LC/MS-MS | 0.01 |
| Imazalil | LC/MS-MS | 0.01 |
| Imazamox | LC/MS-MS | 0.01 |
| Imazaquin | LC/MS-MS | 0.01 |
| Imidachlopride | LC/MS-MS | 0.01 |
| Indoxacarb | LC/MS-MS | 0.01 |
| Iodofenphos | GC/MS-MS | 0.01 |
| Iodosulfuron-methyl | LC/MS-MS | 0.01 |
| Ioxynil | LC/MS-MS | 0.01 |
| Ipconazole | LC/MS-MS | 0.01 |
| Iprodione | GC/MS-MS | 0.01 |
| Iprovalicarbe | LC/MS-MS | 0.01 |
| Isazofos | LC/MS-MS | 0.01 |
| Isobenzan | GC/MS-MS | 0.01 |
| Isocarbophos | LC/MS-MS | 0.01 |
| Isodrine | GC/MS-MS | 0.01 |
| Isofenphos-ethyl | GC/MS-MS | 0.01 |
| Isofenphos-methyl | GC/MS-MS | 0.01 |
| Isoprocarb | LC/MS-MS | 0.01 |
| Isopropaline | LC/MS-MS | 0.01 |
| Isoprothiolane | LC/MS-MS | 0.01 |
| Isoproturon | LC/MS-MS | 0.01 |
| Isopyrazam | LC/MS-MS | 0.01 |
| Isoxaben | LC/MS-MS | 0.01 |
| Isoxadifen-ethyl | GC/MS-MS | 0.01 |
| Isoxaflutole | GC/MS-MS | 0.01 |
| Isoxathion | LC/MS-MS | 0.01 |
| Kresoxim-methyl | LC/MS-MS | 0.01 |
| lambda-Cyhalothrine | GC/MS-MS | 0.01 |
| Lenacil | LC/MS-MS | 0.01 |
| Linuron | LC/MS-MS | 0.01 |
| Lufenurone | LC/MS-MS | 0.01 |
| Malathion(+Malaoxon) | GC/MS-MS | 0.01 |
| Mandipropamide | LC/MS-MS | 0.01 |
| MCPA+MCPB | LC/MS-MS | 0.01 |
| Mecarbam | LC/MS-MS | 0.01 |
| Mecoprop(+Mecoprop-p) | LC/MS-MS | 0.01 |
| Mefenacet | LC/MS-MS | 0.01 |
| Mepanipyrim | GC/MS-MS | 0.01 |
| Mepronil | GC/MS-MS | 0.01 |
| Mesosulfuron-methyl | LC/MS-MS | 0.01 |
| Metaflumizone | LC/MS-MS | 0.01 |
| Metalaxyl(+Metalaxyl-M) | GC/MS-MS | 0.01 |
| Metaldehyde | LC/MS-MS | 0.01 |
| Metamitron | LC/MS-MS | 0.01 |
| Metazachlor | GC/MS-MS | 0.01 |
| Metconazole | LC/MS-MS | 0.01 |
| Methabenzthiazuron | LC/MS-MS | 0.01 |
| Methacrifos | GC/MS-MS | 0.01 |
| Methamidophos | GC/MS-MS | 0.01 |
| Methidathion | GC/MS-MS | 0.01 |
| Methiocarbe | GC/MS-MS | 0.01 |
| Methiocarbe-sulfoxide(+sulfone) | LC/MS-MS | 0.01 |
| Methomyl(+Thiodicarb) | LC/MS-MS | 0.01 |
| Methoxychlore | GC/MS-MS | 0.01 |
| Methoxyfenozide | LC/MS-MS | 0.01 |
| Metobromuron | LC/MS-MS | 0.01 |
| Metolachlore(+S-Metolachlor) | GC/MS-MS | 0.01 |
| Metoxuron | LC/MS-MS | 0.01 |
| Metrafenone | LC/MS-MS | 0.01 |
| Metribuzine | GC/MS-MS | 0.01 |
| Metsulfuron-methyl | LC/MS-MS | 0.01 |
| Mevinphos | GC/MS-MS | 0.01 |
| Milbemectin A4 | LC/MS-MS | 0.01 |
| Mirex | GC/MS-MS | 0.01 |
| Molinate | LC/MS-MS | 0.01 |
| Monalide | GC/MS-MS | 0.01 |
| Monocrotophos | GC/MS-MS | 0.01 |
| Monolinuron | LC/MS-MS | 0.01 |
| Monuron | LC/MS-MS | 0.01 |
| Myclobutanil | GC/MS-MS | 0.01 |
| NAD(1-naphtyl acetamide) | LC/MS-MS | 0.01 |
| Napropamide | GC/MS-MS | 0.01 |
| Neburon | LC/MS-MS | 0.01 |
| Nicosulfuron | LC/MS-MS | 0.01 |
| Nitenpyram | LC/MS-MS | 0.01 |
| Nitrofene | GC/MS-MS | 0.01 |
| Nitrothal-isopropyle | GC/MS-MS | 0.01 |
| Norflurazon | GC/MS-MS | 0.01 |
| Novaluron | LC/MS-MS | 0.01 |
| Nuarimol | LC/MS-MS | 0.01 |
| Orthosulfamuron | LC/MS-MS | 0.01 |
| Oryzalin | LC/MS-MS | 0.01 |
| Oxadiazon | GC/MS-MS | 0.01 |
| Oxadixyl | GC/MS-MS | 0.01 |
| Oxamyl | LC/MS-MS | 0.01 |
| Oxasulfuron | LC/MS-MS | 0.01 |
| Oxyfluorfene | GC/MS-MS | 0.01 |
| Paclobutrazol | LC/MS-MS | 0.01 |
| Paraoxon-ethyl | LC/MS-MS | 0.01 |
| Parathion-ethyl | GC/MS-MS | 0.01 |
| Parathion-methyl | GC/MS-MS | 0.01 |
| PCB101 | GC/MS-MS | 0.01 |
| PCB118 | GC/MS-MS | 0.01 |
| PCB138 | GC/MS-MS | 0.01 |
| PCB153 | GC/MS-MS | 0.01 |
| PCB180 | GC/MS-MS | 0.01 |
| PCB28 | GC/MS-MS | 0.01 |
| PCB52 | GC/MS-MS | 0.01 |
| Penconazole | GC/MS-MS | 0.01 |
| Pencycuron | LC/MS-MS | 0.01 |
| Pendimethaline | GC/MS-MS | 0.01 |
| Penoxsulame | LC/MS-MS | 0.01 |
| Pentachloroanisole | GC/MS-MS | 0.01 |
| Permethrine(cis+trans) | GC/MS-MS | 0.01 |
| Perthane | GC/MS-MS | 0.01 |
| Phenmediphame | LC/MS-MS | 0.01 |
| Phenothrine | GC/MS-MS | 0.01 |
| Phenthoate | GC/MS-MS | 0.01 |
| Phorate(+sulfone) | GC/MS-MS | 0.01 |
| Phosalone | GC/MS-MS | 0.01 |
| Phosmet(+oxon) | LC/MS-MS | 0.01 |
| Phosphamidon | LC/MS-MS | 0.01 |
| Phoxim | LC/MS-MS | 0.01 |
| Picloram | LC/MS-MS | 0.01 |
| Picolinafen | LC/MS-MS | 0.01 |
| Picoxystrobine | LC/MS-MS | 0.01 |
| Pinoxadene | LC/MS-MS | 0.01 |
| Piperonyl-butoxide | GC/MS-MS | 0.01 |
| Pirimicarb | GC/MS-MS | 0.01 |
| Pirimicarb-desmethyl | LC/MS-MS | 0.01 |
| Pirimiphos-ethyl | GC/MS-MS | 0.01 |
| Pirimiphos-methyl | GC/MS-MS | 0.01 |
| Plifenate | GC/MS-MS | 0.01 |
| Pretilachlore | GC/MS-MS | 0.01 |
| Prochloraz(+TCP) | GC/MS-MS | 0.01 |
| Procymidone | GC/MS-MS | 0.01 |

| Compound | Method | LoQ (mg/kg) |
|---|---|---|
| Profenophos | GC/MS-MS | 0.01 |
| Prometryn | GC/MS-MS | 0.01 |
| Propachlore | GC/MS-MS | 0.01 |
| Propamocarbe | LC/MS-MS | 0.01 |
| Propaquizafop | LC/MS-MS | 0.01 |
| Propargite | LC/MS-MS | 0.01 |
| Propazine | GC/MS-MS | 0.01 |
| Propetamphos | GC/MS-MS | 0.01 |
| Prophame | GC/MS-MS | 0.01 |
| Propiconazole | GC/MS-MS | 0.01 |
| Propoxur | LC/MS-MS | 0.01 |
| Propoxycarbazone | LC/MS-MS | 0.01 |
| Propyzamide | GC/MS-MS | 0.01 |
| Proquinazid | GC/MS-MS | 0.01 |
| Prosulfocarbe | GC/MS-MS | 0.01 |
| Prosulfuron | LC/MS-MS | 0.01 |
| Prothioconazole | LC/MS-MS | 0.01 |
| Prothioconazole-desthio | LC/MS-MS | 0.01 |
| Prothiophos | GC/MS-MS | 0.01 |
| Prothoate | GC/MS-MS | 0.01 |
| Pymetrozine | LC/MS-MS | 0.01 |
| Pyraclostrobine | LC/MS-MS | 0.01 |
| Pyraflufen-ethyl | LC/MS-MS | 0.01 |
| Pyrazophos | GC/MS-MS | 0.01 |
| Pyridaben | GC/MS-MS | 0.01 |
| Pyridalyl | GC/MS-MS | 0.01 |
| Pyridaphenthion | GC/MS-MS | 0.01 |
| Pyridate(+metabolite) | LC/MS-MS | 0.01 |
| Pyrifenox | GC/MS-MS | 0.01 |
| Pyrimethanil | GC/MS-MS | 0.01 |
| Pyriproxyfen | GC/MS-MS | 0.01 |
| Pyroxsulam | LC/MS-MS | 0.01 |
| Quinalphos | GC/MS-MS | 0.01 |
| Quinmerac | LC/MS-MS | 0.01 |
| Quinomethionate | GC/MS-MS | 0.01 |
| Quinoxyfen | GC/MS-MS | 0.01 |
| Quintozene(+PCA) | GC/MS-MS | 0.01 |
| Quizalofop(free acid) | LC/MS-MS | 0.01 |
| Quizalofop-ethyl | GC/MS-MS | 0.01 |
| Resmethrine | GC/MS-MS | 0.01 |
| Rimsulfuron | LC/MS-MS | 0.01 |
| Rotenone | LC/MS-MS | 0.01 |
| S421 | GC/MS-MS | 0.01 |
| Sebuthylazine | GC/MS-MS | 0.01 |
| Secbumeton | GC/MS-MS | 0.01 |
| Silthiofam | LC/MS-MS | 0.01 |
| Simazine | LC/MS-MS | 0.01 |
| Spinetoram(Σisomers) | LC/MS-MS | 0.01 |
| Spinosad(Σisomers) | LC/MS-MS | 0.01 |
| Spirodiclofen | LC/MS-MS | 0.01 |
| Spiromesifen | LC/MS-MS | 0.01 |
| Spirotetramate(+4 metabolites) | LC/MS-MS | 0.01 |
| Spiroxamine | LC/MS-MS | 0.01 |
| Sulfosulfuron | LC/MS-MS | 0.01 |
| Sulfotep | GC/MS-MS | 0.01 |
| Sulprofos | GC/MS-MS | 0.01 |
| TCMTB | LC/MS-MS | 0.01 |
| Tebuconazole | GC/MS-MS | 0.01 |
| Tebufenozide | LC/MS-MS | 0.01 |
| Tebufenpyrad | GC/MS-MS | 0.01 |
| Tebupirimphos | GC/MS-MS | 0.01 |
| Tebutam | LC/MS-MS | 0.01 |
| Tecnazene | GC/MS-MS | 0.01 |
| Teflubenzuron | LC/MS-MS | 0.01 |
| Tefluthrine | GC/MS-MS | 0.01 |
| Tepraloxydim | LC/MS-MS | 0.01 |
| Terbacil | GC/MS-MS | 0.01 |
| Terbufos | GC/MS-MS | 0.01 |
| Terbufos-sulfone(+sulfoxide) | LC/MS-MS | 0.01 |
| Terbumeton | LC/MS-MS | 0.01 |
| Terbuthylazine | GC/MS-MS | 0.01 |
| Terbutryne | GC/MS-MS | 0.01 |
| Tetrachlorvinphos | GC/MS-MS | 0.01 |
| Tetraconazole | LC/MS-MS | 0.01 |
| Tetradifon | GC/MS-MS | 0.01 |
| Tetramethrine | GC/MS-MS | 0.01 |
| Tetrasul | GC/MS-MS | 0.01 |
| Thiabendazole | LC/MS-MS | 0.01 |
| Thiachlopride | LC/MS-MS | 0.01 |
| Thiamethoxam(+Clothianidine) | LC/MS-MS | 0.01 |
| Thiencarbazone-methyl | LC/MS-MS | 0.01 |
| Thifensulfuron-methyl | LC/MS-MS | 0.01 |
| Thiobencarb | LC/MS-MS | 0.01 |
| Thiophanate-methyl | LC/MS-MS | 0.01 |
| Thirame | LC/MS-MS | 0.01 |
| TNFA | LC/MS-MS | 0.01 |
| TNFG | LC/MS-MS | 0.05 |
| Tolclofos-methyl | GC/MS-MS | 0.01 |
| Tolylfluanid | GC/MS-MS | 0.01 |
| Tralomethrine | GC/MS-MS | 0.01 |
| Transfluthrine | GC/MS-MS | 0.01 |
| Triadimefone+Triadimenol | GC/MS-MS | 0.01 |
| Triallate | GC/MS-MS | 0.01 |
| Triamiphos | GC/MS-MS | 0.01 |
| Triazamate | LC/MS-MS | 0.01 |
| Triazophos | GC/MS-MS | 0.01 |
| Trichlorfon | LC/MS-MS | 0.01 |
| Trichloronat | GC/MS-MS | 0.01 |
| Triclopyr | LC/MS-MS | 0.01 |
| Tricyclazole | LC/MS-MS | 0.01 |
| Tridemorphe | LC/MS-MS | 0.01 |
| Trifloxystrobine | LC/MS-MS | 0.01 |
| Trifloxysulfuron | LC/MS-MS | 0.01 |
| Triflumizole | LC/MS-MS | 0.01 |
| Triflumuron | LC/MS-MS | 0.01 |
| Trifluraline | GC/MS-MS | 0.01 |
| Triflusulfuron-methyl | LC/MS-MS | 0.01 |
| Triforine | LC/MS-MS | 0.01 |
| Triticonazole | LC/MS-MS | 0.01 |
| Tritosulfuron | LC/MS-MS | 0.01 |
| Valifenalate | GC/MS-MS | 0.01 |
| Vamidothion | LC/MS-MS | 0.01 |
| Vinclozoline(+3,5-dichloroaniline) | GC/MS-MS | 0.01 |
| Warfarin | LC/MS-MS | 0.01 |
| Zoxamide | GC/MS-MS | 0.01 |
