# Supplementary material for: Residues of plant protection products in grey partridge eggs in French cereal ecosystems
Source: Environ Sci Pollut Res Int. 2016 Feb 3;23:9559–73. doi: 10.1007/s11356-016-6093-7 (PMC4871908; doi:10.1007/s11356-016-6093-7)
Supplement: Supplementary file 2 — Online Resource 2 Active substances (ASs, n = 179) listed as used in intensively cultivated farmlands in north-central France in spring and summer 2010–2011, statistics of use, potential exposure, and number of clutches where the AS was detected. Figures into brackets refer to the number of clutches where the AS was detected with no potential exposure of the clutch identified with the method used by Bro et al. (2015). The other compounds detected in the clutches are also provided in the list. Compounds are arranged alphabetically (PDF 13 kb) [file 11356_2016_6093_MOESM2_ESM.pdf]

Active substance	Agricultural use		Potential exposure [a]	Clutches analysed for residues (n=52)	
	% sites (n=12)	% farmers (n=142)	% clutches (n=140)	number of clutches potentially exposed to the AS [a]	number of clutches with the AS detected ("-": *not measured*)
2,4-D	25.0	3.5	1.4	0	0
2,4-DB	8.3	0.7	0.0	0	0
Abamectin	8.3	1.4	1.4	0	0
Acetamiprid	8.3	0.7	0.0	0	0
Acetochlor	41.7	4.9	0.0	0	0
Aclonifen	100.0	36.6	1.4	0	0
Amidosulfuron	33.3	9.2	1.4	0	0
Amitrole	8.3	1.4	0.0	0	-
Anthraquinone	33.3	12.7	0.0	0	0
Asulam	25.0	2.8	1.4	0	-
Azoxystrobin	83.3	38.0	9.3	6	0
Benalaxyl	8.3	1.4	0.0	0	0
Benfluralin	25.0	2.8	0.7	0	0
Benoxacor	16.7	2.8	0.0	0	0
Bentazone	66.7	15.5	0.0	0	0
Benthiavalicarb	8.3	1.4	0.0	0	0
Bifenox	58.3	13.4	1.4	1	0
Bifenthrin	58.3	9.9	0.0	0	0
Boscalid	100.0	57.0	20.7	12	0
Bromoxynil	83.3	24.6	3.6	3	(+1)
Bromuconazole	16.7	1.4	0.7	0	0
Bupirimate	8.3	0.7	0.0	0	0
Carbetamide	16.7	2.1	1.4	0	0
Carfentrazone-ethyl	16.7	2.1	0.0	0	0
Chlorantraniliprole	8.3	0.7	0.0	0	0
Chloridazon	75.0	26.1	4.3	3	0
Chlormequat chloride	100.0	61.3	13.6	9	[b]
Chlorothalonil	100.0	50.0	18.6	8	0
Chlorotoluron	16.7	2.1	0.0	0	0
Chlorpropham	8.3	0.7	0.0	0	0
Chlorpyriphos-ethyl	58.3	12.0	0.7	1	0
Chlorpyriphos-methyl	8.3	0.7	0.0	0	0
Chlorsulfuron	8.3	0.7	0.0	0	0
Clethodim	50.0	13.4	2.1	1	0
Clodinafop-propargyl	50.0	9.9	0.0	0	0
Clomazone	75.0	16.9	0.0	0	0
Clopyralid	91.7	38.7	7.9	3	-
Cloquintocet-mexyl	83.3	25.4	0.0	0	0
Cyazofamid	33.3	8.5	2.1	2	0
Cycloxydim	33.3	3.5	2.9	2	0
Cyfluthrin	50.0	9.2	1.4	1	0
Cymoxanil	58.3	12.7	2.1	1	0
Cypermethrin	100.0	18.3	9.3	6	0
Cyproconazole	100.0	43.0	17.1	11	1 (+1)
Cyprodinil	66.7	11.3	1.4	1	0
DDT(Σisomeres)					(+6)
Deltamethrin	66.7	26.1	7.1	3	0
Desmedipham	75.0	17.6	5.0	3	0
Dicamba	41.7	7.0	0.7	1	0
Dichlorprop-P	16.7	1.4	0.0	0	0
Diclofop-methyl	75.0	23.2	0.0	0	0
Difenoconazole	66.7	23.9	0.7	0	(+1)
Diflufenican	66.7	23.2	0.7	1	(+1)
Dimethachlor	50.0	7.0	0.0	0	0
Dimethenamid-P	33.3	2.8	0.7	0	0
Dimethomorph	41.7	7.7	0.7	0	0
Dinocap	8.3	1.4	0.7	0	0
Diphenylamine					(+3)
Diquat	8.3	2.1	0.0	0	-
Epoxiconazole	100.0	71.1	30.0	13	0
Esfenvalerate	33.3	2.8	0.7	1	0
Ethephon	75.0	35.9	12.1	4	-

[a] with the method used by Bro et al. (2015)

[b] 0 out of 6 clutches analysed using a specific analysis - the SA was detected in none

Active substance	Agricultural use		Potential exposure [a]	Clutches analysed for residues (n=52)	
	% sites (n=12)	% farmers (n=142)	% clutches (n=140)	number of clutches potentially exposed to the AS [a]	number of clutches with the AS detected ("-": *not measured*)
Ethofumesate	75.0	42.3	10.0	6	0
Ethoprophos	8.3	1.4	0.0	0	0
Fenamidone	16.7	1.4	0.0	0	0
Fenazaquin	8.3	0.7	0.0	0	0
Fenoxaprop-P-ethyl	83.3	22.5	0.0	0	0
Fenpropidin	75.0	38.0	10.7	4	3
Fenpropimorph	75.0	19.0	2.9	0	0
Fipronil(+sulfone)					(+3)
Flonicamid	33.3	5.6	2.1	1	0
Florasulam	91.7	31.0	0.7	1	0
Fluazifop-P-butyl	25.0	2.8	0.7	0	0
Fluazinam	41.7	9.2	2.1	1	0
Fludioxonil	50.0	21.8	2.1	0	0
Fluopicolide	16.7	2.8	1.4	1	0
Fluoxastrobin	75.0	40.8	17.9	6	0
Flupyrsulfuron-methyl-sodium	25.0	4.9	0.0	0	-
Flurochloridone	33.3	4.2	0.0	0	0
Fluroxypyr	100.0	44.4	10.0	5	0
Flurtamone	8.3	1.4	0.0	0	0
Flusilazole	25.0	7.7	0.7	0	0
Fluthiamide	33.3	4.9	1.4	1	-
Flutolanil	33.3	5.6	0.0	0	0
Flutriafol	25.0	4.2	0.0	0	0
Foramsulfuron	25.0	4.2	1.4	0	0
Fosetyl-aluminium	8.3	1.4	0.0	0	-
Glufosinate-ammonium	41.7	8.5	1.4	0	-
Glyphosate	75.0	14.1	0.0	0	-
HCH(α,β,δ)					(+1)
Heptachlor(+epoxyde)					(+3)
Hexaconazole	8.3	0.7	0.0	0	0
Hexythiazox	8.3	0.7	0.7	0	0
Imazamox	75.0	16.9	0.0	0	0
Imazaquin	41.7	10.6	0.7	1	0
Imidacloprid	58.3	19.7	0.0	0	0
Iodosulfuron	100.0	53.5	2.1	1	0
Ioxynil	75.0	26.1	2.9	0	0
Iprodione	25.0	2.8	1.4	0	0
Iprovalicarb	8.3	0.7	0.0	0	0
Isoproturon	50.0	16.2	0.0	0	0
Isoxaben	8.3	1.4	0.7	0	0
Isoxadifen-ethyl	50.0	7.0	1.4	1	0
Isoxaflutole	33.3	3.5	0.0	0	0
Kresoxim-methyl	41.7	4.9	0.0	0	0
lambda-Cyhalothrin	91.7	52.8	18.6	9	1
Lenacil	75.0	43.7	9.3	5	0
Linuron	33.3	3.5	0.7	0	0
Maleic hydrazide	16.7	1.4	0.0	0	-
Mancozeb	75.0	18.3	6.4	3	-
Mandipropamid	25.0	5.6	1.4	1	0
Maneb	16.7	3.5	0.7	1	-
MCPA	75.0	33.1	7.1	3	0
Mecoprop-P	66.7	19.0	1.4	1	0
Mefenpyr-diethyl	91.7	37.3	2.1	1	-
Mepiquat chloride	83.3	21.1	2.9	1	-
Mesosulfuron-methyl	100.0	49.3	0.7	1	0
Mesotrione	83.3	25.4	2.1	1	-
Metalaxyl-M	75.0	15.5	0.7	1	0
Metaldehyde	16.7	1.4	0.0	0	0
Metamitron	75.0	45.1	10.0	7	0
Metazachlor	66.7	8.5	0.0	0	0
Metconazole	91.7	38.7	12.1	5	0
Methiocarb	33.3	4.2	0.0	0	0
Metiram	8.3	0.7	0.0	0	-
Metosulam	25.0	4.2	1.4	1	-

Active substance	Agricultural use		Potential exposure [(a)]	Clutches analysed for residues (n=52)	
	% sites (n=12)	% farmers (n=142)	% clutches (n=140)	number of clutches potentially exposed to the AS [(a)]	number of clutches with the AS detected (*"-": not measured*)
Metrafenone	33.3	11.3	3.6	0	0
Metribuzin	66.7	15.5	2.9	0	0
Metsulfuron-methyl	75.0	20.4	0.7	0	0
Myclobutanil	8.3	0.7	0.0	0	0
Napropamide	41.7	8.5	0.0	0	0
Nicosulfuron	75.0	21.1	2.1	2	0
Oxadiargyl	16.7	3.5	0.0	0	-
Oxadiazon	8.3	0.7	0.0	0	0
Paclobutrazol	75.0	9.2	0.0	0	0
PCB153					(+3)
PCB180					(+1)
Penconazole	8.3	1.4	1.4	0	0
Pencycuron	33.3	3.5	0.7	0	0
Pendimethalin	100.0	26.1	0.0	0	0
Phenmedipham	75.0	46.5	11.4	7	0
Picolinafen	8.3	1.4	0.0	0	0
Picoxystrobin	66.7	17.6	6.4	2	0
Pinoxaden	50.0	9.9	0.0	0	0
Pirimicarb	83.3	23.2	5.0	2	0
Prochloraz	91.7	61.3	18.6	11	2 (+2)
Prohexadione-calcium	66.7	12.0	2.9	1	-
Pronamide-propyzamide	25.0	2.8	1.4	0	0
Propamocarb	25.0	5.6	2.1	2	0
Propiconazole	83.3	43.7	19.3	10	0
Propoxycarbazone-sodium	16.7	2.1	0.0	0	-
Proquinazid	8.3	1.4	0.0	0	0
Prosulfocarb	58.3	12.7	1.4	0	0
Prosulfuron	50.0	7.0	2.1	3	0
Prothioconazole	91.7	72.5	34.3	18	0
Pymetrozine	16.7	1.4	0.0	0	0
Pyraclostrobin	75.0	19.7	7.1	4	0
Pyrimethanil	33.3	4.2	1.4	1	0
Pyroxsulam	50.0	7.0	0.0	0	0
Quinmerac	91.7	21.1	4.3	3	0
Quinoxyfen	8.3	1.4	0.0	0	0
Quizalofop-P-ethyl	50.0	11.3	1.4	0	0
Rimsulfuron	16.7	1.4	0.0	0	0
S-Metolachlor	41.7	13.4	1.4	0	0
Spiroxamine	50.0	17.6	7.1	3	0
Sulcotrione	41.7	16.2	2.1	0	-
Sulfosulfuron	16.7	1.4	0.0	0	0
Tau-Fluvalinate	75.0	16.2	2.1	2	0
Tebuconazole	91.7	46.5	17.1	15	1
Tefluthrin	50.0	25.4	0.0	0	0
Tembotrione	33.3	4.9	1.4	2	-
Tetraconazole	33.3	4.2	1.4	0	0
Thiacloprid	66.7	18.3	5.0	1	0
Thiamethoxam	41.7	7.0	0.0	0	0 (+3)
Thifensulfuron-methyl	66.7	19.0	0.0	0	0
Thiophanate	16.7	1.4	0.0	0	-
Thiophanate-methyl	25.0	2.8	2.1	0	0
Thiram	25.0	2.8	0.0	0	0
Triadimenol	16.7	1.4	0.7	1	0
Triallate	16.7	1.4	0.0	0	0
Tribenuron-methyl	66.7	19.0	0.0	0	-
Trifloxystrobin	75.0	21.8	7.9	4	0
Triflusulfuron-methyl	58.3	25.4	3.6	2	0
Trinexapac-ethyl	66.7	30.3	4.3	1	0
Triticonazole	8.3	1.4	0.0	0	0
Zoxamide	25.0	3.5	0.0	0	0
