# Supplementary material for: Residues of plant protection products in grey partridge eggs in French cereal ecosystems
Source: Environ Sci Pollut Res Int. 2016 Feb 3;23:9559–73. doi: 10.1007/s11356-016-6093-7 (PMC4871908; doi:10.1007/s11356-016-6093-7)
Supplement: Supplementary file 3 — Online Resource 3 Compounds detected in the 24 clutches out of the 52 clutches analysed for pesticide residues, and treatments that are likely to have caused the exposure (uses in 2010–2011). Lines separate the different clutches (PDF 18 kb) [file 11356_2016_6093_MOESM3_ESM.pdf]

Clutch code	number of samples	number of eggs analysed	potential exposure: number of ASs[a]	Residue analyses			Potential exposure identified through known farming practices						
				number of ASs measured	compound	concen-tration (mg/kg)	potential exposure identified [a]	period of exposure	treatment	dose (g/ha)	tank mixture	trade co-formulation	other uses in the site
2011-45-24-2	3	8	0	-	Thiamethoxam(+clothianidin)	0.013	no	-	-	-	-	-	thiamethoxam: sugar beets in March (seed treatment, 60 g/ha) maize in April (seed treatment, 69.3 g/ha)
2011-45-583-2	3	8	14	12	Heptachlor(+epoxyde)	< 0.01	no	-	-	-	-	-	-
2011-77P-723-1	2	3	11	7	Heptachlor(+epoxyde)	0.11	no	-	-	-	-	-	-
					Prochloraz(+TCP)	0.041	yes	egg-laying	winter wheat, 3 fields, 1 treatment, 5 & 12 May, spraying	112.5-225.5	yes (with boscalid & epoxiconazole)	no	-
					Bromoxynil	< 0,01	no	-	-	-	-	-	maize in May (112.5 g/ha)
2011-77J-1158-1	1	1	6	6	Prochloraz(+TCP)	0.024	yes	pre-laying & egg-laying	winter wheat, 2 fields, 1 treatment, 10 & 12 May, spraying	162, 267	yes (with epoxiconazole & alpha-cypermethrin)	yes (with tebuconazole)	-
2011-41-1101-1	2	3	0	-	DDT(Σ isomers)	0.046	no	-	-	-	-	-	-
2011-76-952-1	2	3	18	18	Fipronil(+sulfone)	0.0085	no	-	-	-	-	-	-
					HCH(α+β+δ)	0.015	no	-	-	-	-	-	-
2011-27-1040-1	1	1	19	15	Cyproconazole	0.021	yes	pre-laying	winter wheat, 1 field, 1 treatment, 18 April, spraying	64	no	yes (with propiconazole)	rapeseed in early May (80 g/ha)
2011-45-586-1	2	4	9	9	Cyhalothrin(lambda)	< 0.01	yes	egg-laying	peas, 3 treatments, April-May, spraying	6	no	yes (with pyrimicarbe)	rapeseed in March-April (7 g/ha) winter wheat in late May - early June (3.5 g/ha) sugar beets in early June (7.25 g/ha) potatoes in late June (11 g/ha)
					Fenpropidin	0.34	yes	egg-laying	spring barley, 1 field, 1 treatment, 2 May, spraying	225	no	yes (with tebuconazole & propiconazole)	spring barley/wheat in April-May (187-300 g/ha) sugar beets in August (375-412 g/ha)
					Heptachlor(+epoxyde)	< 0.01	no	-	-	-	-	-	-
					Tebuconazole	< 0.01	yes	egg-laying	spring barley, 1 field, 1 treatment, 2 May, spraying	75	no	yes (with propiconazole & fenpropidine)	winter wheat in April-May (75-198.7 g/ha) rapeseed in March-April (100-250 g/ha)
2011-45-587-1	2	6	4	4	Diphenylamine	0.01	no	-	-	-	-	-	-
					Fenpropidin	0.036	yes	pre-laying	spring barley, 1 field, 1 treatment, 2nd May, spraying	225	no	yes (with propiconazole & fenpropidine)	spring barley/wheat in April-May (187-300 g/ha) sugar beets in August (375-412 g/ha)
2011-80-871-2	3	6	7	7	Fipronil(+sulfone)	0.0083	no	-	-	-	-	-	-
					PCB153	< 0.01	no	-	-	-	-	-	-
					Thiamethoxam(+clothianidin)	0.037	no	-	-	-	-	-	thiamethoxam: maize in April (seed treatment, 69.3 g/ha)
					of which Clothianidin	0.032	no	-	-	-	-	-	-
2011-51-145-2	1	2	0	-	Diphenylamine	< 0.01	no	-	-	-	-	-	-
2011-41-1101-2	1	2	0	-	DDT(Σ isomers)	< 0.01	no	-	-	-	-	-	-
2011-41-1073-1	1	1	.	-	DDT(Σ isomers)	< 0.01	no	-	-	-	-	-	-
2011-41-1075-1	1	3	.	-	DDT(Σ isomers)	0.027	no	-	-	-	-	-	-
2011-41-1107-1	1	2	0	-	DDT(Σ isomers)	0.03	no	-	-	-	-	-	-
2011-45-579-1	1	2	.	-	Cyproconazole	0.015	no	-	-	-	-	-	winter wheat in late April - early May (64 g/ha) spring barley in late May (52.8 g/ha)
2011-45-658-1	1	1	0	-	DDT(Σ isomers)	0.016	no	-	-	-	-	-	-

[a] with the method used by Bro et al. (2015)

Clutch code	number of samples	number of eggs analysed	potential exposure: number of ASs[a]	Residue analyses			Potential exposure identified through known farming practices						
				number of ASs measured	compound	concen-tration (mg/kg)	potential exposure identified [a]	period of exposure	treatment	dose (g/ha)	tank mixture	trade co-formulation	other uses in the site
2011-59-841-1	1	3	.	-	Fipronil(+sulfone)	0.0068	no	-	-	-	-	-	-
					PCB153	< 0.01	no	-	-	-	-	-	-
					PCB180	< 0.01	no	-	-	-	-	-	-
					Thiamethoxam(+clothianidin)	0.067	no	-	-	-	-	-	thiamethoxam: peas for canning in May (seed treatment, 116.7 g/ha)
					of which Clothianidin	0.057	no	-	-	-	.	.	.
2011-59-803-1	1	3	.	-	Prochloraz(+TCP)	< 0.01	no	-	-	-	-	-	winter wheat in April - May (450-598 g/ha)
2011-80-906-1	2	3	3	3	Prochloraz(+TCP)	< 0.01	no	-	-	-			winter winter in May-June (225-270 g/ha)
2011-80-886-1	1	2	8	8	Diflufenican	0.016	no	-	-	-	-	-	spring & winter barley in late March (62.6 g/l)
2011-80-917-1	1	1	.	-	PCB153	< 0.01	no	-	-	-	-	-	-
2011-51-111-1	1	2	11	8	Fenpropidin	0.032	yes	pre-laying & egg-laying	winter wheat & spring barley, 2 fields, 2 treatments, late April - early May, spraying	50-250	yes (winter wheat: with epoxiconazole & fluroxypyr & mancozeb; spring barley: with boscalid & epoxiconazole)	yes (with propiconazole)	sugar beets in July (262.5 g/ha)
2011-76-958-2	1	1	5	5	Difenoconazole	0.013	no	-	-	-	-	-	flax in May (125 g/ha) sugar beets in July-August (80-100 g/ha)
					Diphenylamine	0.019	no	-	-	-	-	-	-
