# Supplementary material for: Residues of plant protection products in grey partridge eggs in French cereal ecosystems
Source: Environ Sci Pollut Res Int. 2016 Feb 3;23:9559–73. doi: 10.1007/s11356-016-6093-7 (PMC4871908; doi:10.1007/s11356-016-6093-7)
Supplement: Supplementary file 4 — Online Resource 4 Information about compounds that were detected in partridge’ eggs. The dash “−” indicates that the information was not found. (PDF 41.4 kb) [file 11356_2016_6093_MOESM4_ESM.pdf]

Compound	Physico-chemical properties [1,2]			Behaviour in the environment [1,2]		Metabolism in laying hens [f]				MLR [5, g]	Ecotoxicity			Long-term risk assessment for bird species		
	H [a]	Photo-lysis DT_{50} [b]	LogP [c]	Soil DT_{50} [d]	Bio degra-dation [e]	excretion rate	main residues detected in eggs	transfer rate, plateau concentration	Refe-rence [2, 3, 4]		LD_{50} [1,2, h]	LC_{50} [1,2, i]	NOEL [6, j]	first-tier TER_{lt} [k]	higher-tier TER_{lt} [l]	Refe-rence [6]
Bromoxynil *data for phenol*	$5.3\ 10^{-4}$ (calc)	< 1 (pH: 5-7.5)	1.04-1.3 (pH:2-7, 25°C) (octanoate: 5.9 (pH=7, 25°C))	1-8	NEB	"approximately 70-80 % of the AR"	bromoxynil phenol (parent compound in the exp.)	"transfer of residues to eggs, at these rates [5.3 mg/kg bw per d during 7 days], are relatively low"	EFSA (2012a)	0.05	217 (CV)	2080 (CV) 1380 (AP)	-	-	-	-
Clothianidin	$2.9\ 10^{-11}$ (20°C)	0.14 (pH=7)	0.87-0.90 (pH=4-10, 25°C)	13-660	NEB	-	"the major residue in poultry eggs consisted of thiazolylnitroguanidine TZNG (88 %), parent was found at 21 % of the TRR"	"residue levels in eggs increased from 0.38-0.94 mg eq/kg at 24 to 53 hours after the 1st dose" (laying hens dosed with 10 mg/kg bw per d of clothianidin during 3 days)	EFSA (2014a)	0.01	430 (CCJ)	> 5000 (CV)	56.8 (CV) (ANSES 2011)	> 25	/	ANSES (2011)
Cyproconazole	$5\ 10^{-5}$	>40 (pH=7, natural sunlight)	3.09 (pH=7.2, 25°C)	62.1-501.2	NEB	-	- Cyproconazole: 10–30% TTR (white), 22–50% TTR (yolk)<br>- NOA421153 (M9/M14): 35–44% TRR (white), 14–28% TRR (yolks)<br>- NOA408616 (M15): 18–36% TRR (white), 4–10% (yolk)	-	JMPR (2011)	0.05	94 (CV)	468 (CV) 151 (AP)	6.6 (CV) 1.4 (AP)	0.79	skylark: 6.4 yellowhammer : 5.7 yellow wagtail: 5.6	EFSA (2010)
Difenoconazole	$9\ 10^{-7}$ (25°C)	-	4.36 (pH=8, 25°C)	265 (max)	NEB	-	"the metabolite 1,2,4-triazole […] in significant proportions in eggs (32 – 75% TRR)."	plateau:<br>- 168 hrs (yolk)<br>- 120 hrs (white)	EFSA (2011a)	0.05	> 2000 (CCJ)	392 (CV)	9.71 (CV)	0.91	skylark: 5.1 - 287	EFSA (2011a)
Diflufenican	> 1.18 10^{-2}	-	4.2 (20 °C)	224-621	NEB	"the majority of the AR was excreted (85-89 %)"	diflufenican	- "transfer of residues to eggs and tissues is relatively low, […] less than 0.3 % were found in the eggs"<br>- plateau: egg = 8 days	EFSA (2007a, 2013)	0.05	> 2100 (CV)	-	91.84 (-)	25.37	/	EFSA (2007a)
Fenpropidin	10.7 (25°C) (calc)	-	0.83 - 4.5 (pH: 4-9, 25°C)	7-116	NEB	"the majority of the AR was excreted in the urine and faeces (88% to 92%)"	CGA 289267, forming at least 60% of the TRR in eggs	plateau: 72 hrs in eggs	EFSA (2007b, 2011b)	0.02	369 (PC) 431(CV)	> 1400 (CV)	14.6 (NOAE L, CV)	0.65	skylark, yellowhammer : 2.9	EFSA (2007b)
Fipronil(+sulfone) *data for fipronil*	$2.31\ 10^{-4}$	0.33 (pH 5-25)	3.5 (20°C)	33-120 (20°C)	NEB	"a large proportion of the AR was eliminated and recovered in the faeces (ca. 28% to 42%)"	- Fipronil and sulfone<br>- "The sulfone metabolite was also reported to be the major constituent […], accounting for more than 95% of the total radioactivity"	- "the residues observed in milk, egg and animal tissues were found to be strictly linearly related to the residue dose levels in animal feed"<br>- "a plateau level was reached in eggs after 21 days of exposure"<br>- detected days 2 to 12 post-treatment	EFSA (2012b), Kitulagodage et al. (2011a)	0.015	fipronil: 11.3 (CV) sulfone: 41 (CV)	3.77 (CV)	0.88 (CV)	<0.001	-	EFSA (2006)
Lambda-cyhalothrin	$2\ 10^{-2}$ (20°C)	5-13	7 (20°C)	2-40	EB	98%	lambda-cyhalothrin	plateau: residues detected after 7-9d of exposure (yolk)	EFSA (2014b,c)	0.02	>3900 (AP)	>970 (CV)	3.3 (AP)	9.5	/	EFSA (2014c)

Compound	Physico-chemical properties [1,2]			Behaviour in the environment [1,2]		Metabolism in laying hens [f]				MLR [5, g]	Ecotoxicity			Long-term risk assessment for bird species		
	H [a]	Photo-lysis DT_{50} [b]	LogP [c]	Soil DT_{50} [d]	Bio degra-dation [e]	excretion rate	main residues detected in eggs	transfer rate, plateau concentration	Refe-rence [2, 3, 4]		LD_{50} [1,2, h]	LC_{50} [1,2, i]	NOEL [6, j]	first-tier TER_{lt} [k]	higher-tier TER_{lt} [l]	Refe-rence [6]
Prochloraz	1.64 10^{-3}	1.7 (pH=5, 25°C) extrapolated to natural sunlight	3.50-4.40 (pH: 4-9; 23-25°C)	0,4-245	NEB	-	-	"No MRLs were proposed for poultry matrices as it was clear from the metabolism study that significant residues are not expected to be present"	EFSA (2011c)	0.10	662 (CV); 707 (AP)	> 1500 (CV)	-	-	-	EFSA (2011c)
Tebuconazole	1 10^{-7} (20°C) (calc)	590	3.7 (pH=7, 20°C)	20->365	NEB	-	tebuconazole, 1,2,4-triazole	- for one study it is reported that "when laying hens were fed up to the highest dose of tebuconazole (1.26 mg/kg bw/d) for 28 days, no residues of tebuconazole and hydroxy-tebuconazole in eggs were detected"<br>- "In eggs a plateau is reached 2 days after the first dose"	EFSA (2011d, 2014d)	0.10	> 1900 (CV)	> 700 (CV)	5.8 (CV)	≥0.5	1.5 to ≥5.3	EFSA (2014d)
Thiamethoxam	4.7 10^{-10} (25°C)	2.3-3.1 (pH=5)	-0.13 (25 °C)	7-172	NEB	"readily excreted in urine and faeces (61-82 % TRR)" *(hen+goat data)*	- metabolite TZNG (45-59 % TRR)<br>- "parent thiamethoxam was not the major component of the residue"	- equally in yolk and white<br>- 0.29 mg eq/kg for 6.9–7.9 mg/kg bw per d of thiamethoxam during 4 days	EFSA (2014a)	0.01	1552 (CV) 576 (AP)	>1929 (CV) >1175 (AP)	74 (CV) 34.9 (AP) [1]	-	-	-
DDT(Σisomers) *data for DDT*	8.43 10^{-1} (25°C)	-	6.91 (pH=7, 20°C)	4-30 years (Mc Bean 2012)	NEB	low	DDT (97% in the yolk, 3% in the white) and DDE	bioaccumulation	HSDB	0.05	2240 (AP)	-	-	-	-	-
Diphenylamine	0.321 (25°C)	0.2 (pH=7)	3.82 (pH=7, 20°C)	-	-	-	-	"MRLs are not required for poultry tissues and eggs." EFSA (2012c)	EFSA (2012c), HSDB	0.05	> 2250 (CV)	-	-	"Not applicable due to mode of use-indoor use"		EFSA (2012c)
HCH(α,β,δ) *data for γ isomer*	1.48 10^{-6} (25°C)	28 (pH=7)	3.5 (pH=7, 20°C)	148	NEB	-	-	" Only traces of radioactivity were found in the albumen. Chicks contained significantly less 14 C-labelled residues than the eggs from which they were hatched."	EFSA (2005), Saha et al. (1976)	0.01	122 (CV)	-	0.6 EFSA (2005)	-	-	-
Heptachlor (+epoxide) *data for heptachlor*	3.53 10^{2} (25°C)	-	5.44	250	NEB	-	Heptachlor, heptachlor epoxide	accumulation	Kielhorn et al. (2006)	0.02	>2000 (AP)	93 (CCJ) 480 (AP) Kielhorn et al. (2006)	-	-	-	-

(a) H (Henry's law) – Pa m³ / mol. Ability of the AS in solution to volatilize at a given temperature. An AS is considered as volatile when H >> 1.
(b) Photolysis DT_{50} – days. Time taken for 50% of the AS to degrade in water under the influence of light at a given pH.
(c) LogP (P: octanol/water partition coefficient) – dimensionless. Indicator of the lipid solubility of the AS at a given temperature and pH. An AS is considered as liable to bioaccumulate when logP ≥ 3 (AGRITOX).
(d) Soil DT_{50} (field conditions) – days. Time required for 50% of the initial quantity of the AS to dissipate in a given soil type. It includes other degradation pathways.
(e) Biodegradation. NEB: not easily biodegradable, EB: easily biodegradable.
(f) Metabolism in laying hens (experiments using radio-labelled AS). Excretion of an AS and its metabolites mainly occurs through urine and faeces. The faster the excretion, the less concentrated the AS / metabolites, and the less time it has to exert its toxicity. Data should not be compared across compounds since experimental conditions of studies are not similar (dose, duration, etc.); see references for further details. AR: applied radioactivity, TRR: total radioactive residue.
(g) MRL (Maximum Residue Levels) in poultry eggs – mg/kg. MRLs are "the upper legal levels of a concentration for pesticide residues in or on food or feed based on good agricultural practices and to ensure the lowest possible consumer exposure. Regulation (EC) No 396/2005 establishes the MRLs of pesticides permitted in products of plant or animal origin intended for human or animal consumption. Annexes specify the MRLs and the food commodities to which they apply." (http://www.efsa.europa.eu/en/pesticides/mrls).
(h) LD_{50} (acute oral toxicity) – mg of a.s. / kg of body weight of the tested animals. Dose of the AS that is lethal for 50 % of a batch of laboratory animals exposed to a single administration.
(i) LC_{50} – mg a.s. / kg bw / day. Concentration of the AS in the diet that is lethal to 50% of exposed animals. The concentration in the food (mg / kg) is converted to mg / kg of body weight / day.
(j) NO(A)EL (No Observed (Adverse) Effect Level) – mg a.s. / kg bw / day. Maximum amount of an AS whose daily intake during a long-term dietary exposure would have no (adverse) effect on bird reproduction. CV: bobwhite quail (*Colinus virginianus*), AP: mallard (*Anas plathyrhyncos*).
(k) first-tier TER_{lt} (long-term toxicity-to-exposure ratio) – dimensionless. Lowest value of the different scenari for birds (see Bro et al. 2015; EFSA 2009). The screening step is a worst-case model. If an AS and its associated use do not pass the screening step ($TER_{lt} \leq 5$), then a higher-tier risk assessment is estimated using more realistic assumptions.
(l) higher-tier TER_{lt}. Higher-tier refinement model (see EFSA 2009).

Source of data (accessed September-November 2015)
(1) AGRITOX database managed by the French Ministry of Agriculture (http://www.agritox.anses.fr/index.php), Pesticide Properties DataBase (http://sitem.herts.ac.uk/aeru/ppdb/en/index.htm)
(2) HSDB - Hazardous Substances Data Bank http://toxnet.nlm.nih.gov/newtoxnet/hsdb.htm
(3) European Food Safety Authority *reasoned opinions* (http://www.efsa.europa.eu/en/publications.htm)
(4) Joint FAO/WHO Meeting on Pesticide Residues reports (http://apps.who.int/pesticide-residues-jmpr-database or http://www.inchem.org/pages/jmpr.html)
(5) European Union Pesticides database (http://ec.europa.eu/food/plant/pesticides/eu-pesticides-database/public/?event=pesticide.residue.selection&language=EN) for bird egg (code 1030000)
(6) European Food Safety Authority *conclusions* on pesticide (http://www.efsa.europa.eu/en/publications.htm)

References

ANSES (2011) Avis de l'Agence nationale de sécurité sanitaire de l'alimentation, de l'environnement et du travail relatif à une demande de mise sur le marché pour la préparation CHEYENNE, à base de clothianidine, de la société PHILAGRO France. Anses – dossier n°2010-1397 – CHEYENNE, 25p. https://www.anses.fr/fr/system/files/DPR2010ha1397.pdf (In French)

Bro E, Millot F, Decors A, Devillers J (2015) Quantification of potential exposure of grey partridge (*Perdix perdix)* to pesticide active substances in farmlands. Sci Total Environ 521–522:315-325. http://dx.doi.org/10.1016/j.scitotenv.2015.03.073

EFSA – European Food Safety Authority (2014a) Reasoned opinion on the review of the existing maximum residue levels (MRLs) for clothianidin and thiamethoxam according to Article 12 of Regulation (EC) No 396/2005. EFSA Journal 12(12):3918,120p. doi:10.2903/j.efsa.2014.3918

EFSA – European Food Safety Authority(2014b) Reasoned opinion on the review of the existing maximum residue levels (MRLs) for lambda-cyhalothrin according to Article 12 of Regulation (EC) No 396/2005. EFSA Journal 12(1):3546,117p. doi:10.2903/j.efsa.2014.3546

EFSA – European Food Safety Authority (2014c) Conclusion on the peer review of the pesticide risk assessment of the active substance lambda-cyhalothrin. EFSA Journal 12(5):3677,170p. doi:10.2903/j.efsa.2014.3677

EFSA – European Food Safety Authority (2014d) Conclusion on the peer review of the pesticide risk assessment of the active substance tebuconazole. EFSA Journal 12(1):3485,98p. doi:10.2903/j.efsa.2014.3485

EFSA – European Food Safety Authority (2013) Reasoned opinion on the review of the existing maximum residue levels (MRLs) for diflufenican according to Article 12 of Regulation (EC) No 396/2005. EFSA Journal 11(6):3281,42p. doi:10.2903/j.efsa.2013.3281

EFSA – European Food Safety Authority (2012a) Reasoned opinion on the review of the existing maximum residue levels (MRLs) for bromoxynil according to Article 12 of Regulation (EC) No 396/2005. EFSA Journal 10(8):2861,41p. doi:10.2903/j.efsa.2012.2861

EFSA – European Food Safety Authority (2012b) Reasoned opinion on the review of the existing maximum residue levels (MRLs) for fipronil according to Article 12 of Regulation (EC) No 396/2005. EFSA Journal 10(4):2688,44p. doi:10.2903/j.efsa.2012.2688

EFSA – European Food Safety Authority (2012c) Conclusion on the peer review of the pesticide risk assessment of the active substance diphenylamine. EFSA Journal 10(1):2486, 59p. doi:10.2903/j.efsa.2012.2486

EFSA – European Food Safety Authority (2011a) Conclusion on the peer review of the pesticide risk assessment of the active substance difenoconazole. EFSA Journal 9(1):1967,71p. doi:10.2903/j.efsa.2011.1967

EFSA – European Food Safety Authority (2011b) Review of the existing maximum residue levels (MRLs) for fenpropidin according to Article 12 of Regulation (EC) No 396/2005. EFSA Journal 9(8):2333,42p. doi:10.2903/j.efsa.2011.2333

EFSA – European Food Safety Authority (2011c) Conclusion on the peer review of the pesticide risk assessment of the active substance prochloraz. EFSA Journal 9(7):2323,120p. doi:10.2903/j.efsa.2011.2323

EFSA – European Food Safety Authority (2011d) Review of the existing maximum residue levels (MRLs) for tebuconazole according to Article 12 of Regulation (EC) No 396/2005. EFSA Journal 9(8):2339,96 p. doi:10.2903/j.efsa.2011.2339

EFSA – European Food Safety Authority (2010) Conclusion on the peer review of the pesticide risk assessment of the active substance cyproconazole. EFSA Journal 8(11):1897,73p. doi:10.2903/j.efsa.2010.1897

EFSA – European Food Safety Authority (2009) Guidance Document on Risk Assessment for Birds and Mammals on request from EFSA. EFSA Journal 7(12):1438. (revised July 2010) doi:10.2903/j.efsa.2009.1438

EFSA – European Food Safety Authority (2007a) Conclusion regarding the peer review of the pesticide risk assessment of the active substance diflufenican. EFSA Scientific Report 122:1-84. doi:10.2903/j.efsa.2008.122r

EFSA – European Food Safety Authority (2007b) Conclusion regarding the peer review of the pesticide risk assessment of the active substance fenpropidin. EFSA Scientific Report 124:1-84. doi:10.2903/j.efsa.2008.124r

EFSA – European Food Safety Authority (2006) Conclusion regarding the peer review of the pesticide risk assessment of the active substance fipronil. Scientific Report 65:1-110. doi:10.2903/j.efsa.2006.65r

EFSA – European Food Safety Authority (2005) Opinion of the scientific panel on contaminants in the food chain on a request from the Commission related to gamma-HCH and other hexachlorocyclohexanes as undesirable substances in animal feed. EFSA Journal 250:1-39. doi:10.2903/j.efsa.2005.250

HSDB – Hazardous Substances Data Bank http://toxnet.nlm.nih.gov/newtoxnet/hsdb.htm

JMPR – Joint FAO/WHO Meeting on Pesticide Residues (2011) Pesticide residues in food 2010. Report of the Joint Meeting of the FAO Panel of Experts on Pesticide Residues in Food and the Environment and the WHO Core Assessment Group on Pesticide Residues. Rome, Italy, 21–30 September 2010. FAO Plant Production and Protection Paper 167:590p. http://apps.who.int/pesticide-residues-jmpr-database/

Kielhorn J, Schmidt S, Mangelsdorf I, Howe P (2006) HEPTACHLOR. Concise International Chemical Assessment Document 70. http://www.who.int/ipcs/publications/cicad/cicads_alphabetical/en/

Kitulagodage M, Buttemer WA, Astheimer LB (2011) Adverse effects of fipronil on avian reproduction and development: maternal transfer of fipronil to eggs in zebra finch *Taeniopygia guttata* and *in ovo* exposure in chickens *Gallus domesticus*. Ecotoxicology 20:653-660. doi:10.1007/s10646-011-0605-5

MacBean C editor (2012) The pesticide manual – a world compendium. XVIth ed. British Crop Production Council, Alton

Saha JG, Burrage RH (1976) Residues of lindane and its metabolites in eggs, chicks, and body tissues of hen pheasants after ingestion of lindane-^{14}C via treated wheat seed or gelatin capsules. J Environ Sci Health Bull 11:67-93. doi:10.1080/03601237609372026
